# Developmental epigenetic programming by Tet1/3 determines peripheral CD8 T cell fate

**DOI:** 10.1038/s44319-025-00439-z

**Published:** 2025-04-02

**Authors:** Kara M Misel-Wuchter, Andrew L Thurman, Jordan T Johnson, Athmane Teghanemt, Neelam Gautam, Alejandro A Pezzulo, Jennifer R Bermick, Noah S Butler, Priya D Issuree

**Affiliations:** 1 Inflammation Program, University of Iowa, Iowa City, IA USA; 2 Molecular Medicine Graduate Program, University of Iowa, Iowa City, IA USA; 3 Department of Internal Medicine, University of Iowa, Iowa City, IA USA; 4 Immunology Graduate Program, University of Iowa, Iowa City, IA USA; 5 Department of Pediatrics, University of Iowa, Iowa City, IA USA; 6 Department of Microbiology and Immunology, University of Iowa, Iowa City, IA USA; 7 Graduate Program in Molecular Physiology and Biophysics, University of Iowa, Iowa City, IA USA

**Keywords:** DNA Demethylation, Memory CD8 T Cells, Effector CD8 T Cells, T-Cell Development, Epigenetics, Chromatin, Transcription & Genomics, Immunology, Microbiology, Virology & Host Pathogen Interaction

## Abstract

In response to infections, naive CD8 T cells give rise to effector and memory T cells. However, eliciting long-lived memory CD8 T cells remains a challenge for many infections. DNA demethylation of cytosines within CpG dinucleotides by Tet enzymes is a key epigenetic mechanism that regulates short- and long-term transcriptional programs in cells. Currently, their roles in modulating CD8 T-cell effector and memory differentiation are unclear. Here, we report that developing CD8 T cells lacking Tet1/3 preferentially differentiate into short-lived effector and effector memory cells following acute infection. Using genome-wide analyses, mice in which Tet1/3 were ablated during T-cell development and mature CD8 T cells, respectively, we show that Tet1/3 regulates these cell fates by licensing the chromatin landscape of genes downstream of T-cell receptor activation during thymic T-cell maturation. However, in mature CD8 T cells, Tet1/3 are dispensable for effector and memory cell fates. These findings unveil context-specific roles of DNA demethylation, which are essential for defining pathways that contribute to CD8 memory T-cell generation in response to infections.

## Synopsis

CD8 T cells lacking Tet1/3 preferentially differentiate into short-lived effector and effector memory cells following acute infection. Tet1/3 thus act as determinants of CD8 T cell fates by promoting chromatin accessibility at TCR-responsive genes during T cell development.

- Tet1/3 loss predisposes CD8 T cells to adopt short-lived effector and effector memory cell fates during LCMV infection.
- Tet1/3 are dispensable for CD8 effector and memory T cell fate during peripheral T cell priming.
- Tet1/3 license the chromatin landscape of TCR-responsive genes during thymic T cell maturation.
- These genes have regulatory elements enriched for JunB, but JunB binding cannot promote chromatin decompaction.

## Abstract (web summary)

CD8 T cells lacking Tet1/3 preferentially differentiate into short-lived effector and effector memory cells following acute infection. Tet1/3 thus act as determinants of CD8 T cell fates by promoting chromatin accessibility at TCR-responsive genes during T cell development.

## Graphical abstract

**Figure:**
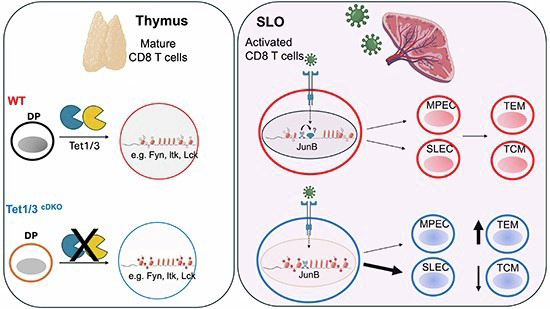

**Figure:**
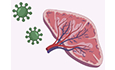

## Introduction

CD8 T cells are uniquely equipped to kill pathogen-infected cells during an infection and are undeterred by point mutations that alter the efficacy of neutralizing antibodies against pathogens. However, eliciting a pool of long-lived functional memory CD8 T cells remains a challenge for many infections (Harty et al, 2000; Sallusto et al, 2010; van de Wall et al, 2021). Understanding the factors and mechanisms that govern the differentiation of distinct memory subsets is the first step in successfully manipulating the immune system for effective and long-term vaccination outcomes.

During an acute infection, a significant portion of proliferating CD8 T cells undergo terminal differentiation into effector cells, commonly known as short-lived effector cells (SLECs). These cells play a crucial role in eliminating the acute infection and typically succumb to apoptosis, although a surviving subset of these effector-like cells can persist into the memory pool with some memory-like features (Milner et al, 2020; Renkema et al, 2020). In contrast, a subset of early-activated cells generates memory precursor effector cells (MPECs), which exhibit multipotency and can differentiate into various types of long-lived memory cells, including central memory (T_CM_) and effector memory (T_EM_) cells (Chung et al, 2021). These distinct memory cell populations possess distinct homing properties and functional capabilities when responding to secondary infections (Gerlach et al, 2013; Masopust et al, 2001; Wherry et al, 2003).

A large body of evidence suggests that multiple signals, including T-cell receptor (TCR), signaling, inflammation, and metabolic signaling, can orchestrate CD8 T-cell fate decisions early during an immune response (Chung et al, 2021). TCR signaling is deemed a key determinant of memory commitment, function, and the diversity of the memory pool (Chin et al, 2022; Daniels and Teixeiro, 2015; Solouki et al, 2020). The integration of TCR signals to alter cell fate outcomes ultimately depends on both the activation of a transcription factor network and concurrent changes in epigenetic modifications that create a chromatin environment permissive for transcription. How epigenetic processes allow for the propagation of upstream TCR signals and/or enable these signals to be fine-tuned to control cell fate outcomes is an area of ongoing investigation.

A key epigenetic change accompanying the differentiation of CD8 T cells involves alterations in DNA methylation at the fifth carbon of cytosines (known as 5-methylcytosine or 5mC), a modification commonly linked to the suppression of transcription (Cedar et al, 2022; Greenberg and Bourc’his, 2019; Jones, 2012). During acute LCMV infection, effector CD8 T cells experience loss of DNA methylation in promoter and enhancer-like regions in genes associated with effector CD8 T-cell function, while cells that give rise to memory cells acquire de novo DNA methylation (Scharer et al, 2013; Youngblood et al, 2017). The de novo methyltransferase Dnmt3a, establishes new methylation marks in genes associated with naive T-cell states such as *Tcf7*, *Cd62l*, and *Ccr7* and is critical for restraining the number of memory precursor effector cells that are generated early during T-cell activation (Ladle et al, 2016; Youngblood et al, 2017).

Meanwhile, the role of DNA demethylation in specifying distinct cell fates during viral infection is less clear. Active DNA demethylation is mediated by the family of Ten-eleven translocation (Tet) methylcytosine dioxygenases, which is comprised of three genes (Tet1, Tet2, and Tet3) that are expressed in T cells (Issuree et al, 2018; Tsagaratou et al, 2017; Wu and Zhang, 2017). To our knowledge, only Tet2 has been examined in the context of memory CD8 T-cell differentiation. Using mice in which Tet2 was conditionally deleted in developing T cells, it was previously shown that loss of Tet2 leads to increased generation of MPEC and T_CM_ CD8 T cells following acute LCMV infection (Carty et al, 2018). The similarity of these phenotypes with Dnmt3a deficiency is striking, given that Dnmt3A catalyzes the de novo methylation of cytosines and Tet2 oxidizes 5mC to 5hmC. The contributions of Tet1 and Tet3 in regulating epigenetic programs during CD8 T-cell differentiation have not been examined.

To shed insights into the role of DNA demethylation on CD8 T-cell outcomes during a viral infection, in this study, we sought to elucidate the roles of Tet1 and Tet3 on CD8 T-cell fate outcomes during acute LCMV infection. We discovered that Tet1/3 were required to restrain the formation of SLECs and favored the differentiation of T_CM_ cells upon acute LCMV infection in a cell-intrinsic manner. Unexpectedly, however, we discovered that modulation of these cell fate programs was not dependent on the activities of Tet1/3 during T-cell priming. By employing genome-wide epigenetic profiling and by utilizing two distinct lines of conditional mice, in which Tet1/3 were ablated during T-cell development and in mature CD8 T cells, respectively, we established the necessity of Tet1/3 during T-cell development. Specifically, Tet1/3 were essential during development to license the chromatin landscape of genes downstream of TCR activation. Tet1/3 supported chromatin accessibility at TCR-responsive regulatory elements enriched in motifs belonging to bZIP, ETS, RUNT, and NFAT transcription factor families, which are known transcriptional regulators for early effector CD8 T-cell differentiation. However, despite reduced chromatin accessibility in Tet1/3-deficient cells, binding of the AP-1 family member JunB, a critical bZIP transcription factor, was intact, suggesting chromatin remodeling is likely required for partnership with other TFs. In contrast, we found that early changes in DNA demethylation following TCR activation did not occur within lineage-defining genes, and Tet1/3 were dispensable for effector and memory CD8 cell fate outcomes in peripheral CD8 T cells. Together, our studies underscore the critical role of Tet1/3 in the early and long-term epigenetic programming of CD8 T cells in the thymus. Most notably, they bring into focus the importance of understanding the unique contributions of Tet enzymes in a context-dependent manner to effectively manipulate epigenetic pathways for optimal vaccination outcomes.

## Results

### Loss of Tet1 and Tet3 in developing CD8 T cells results in increased SLECs and effector memory T-cell differentiation during acute LCMV infection

**Figure 1:**
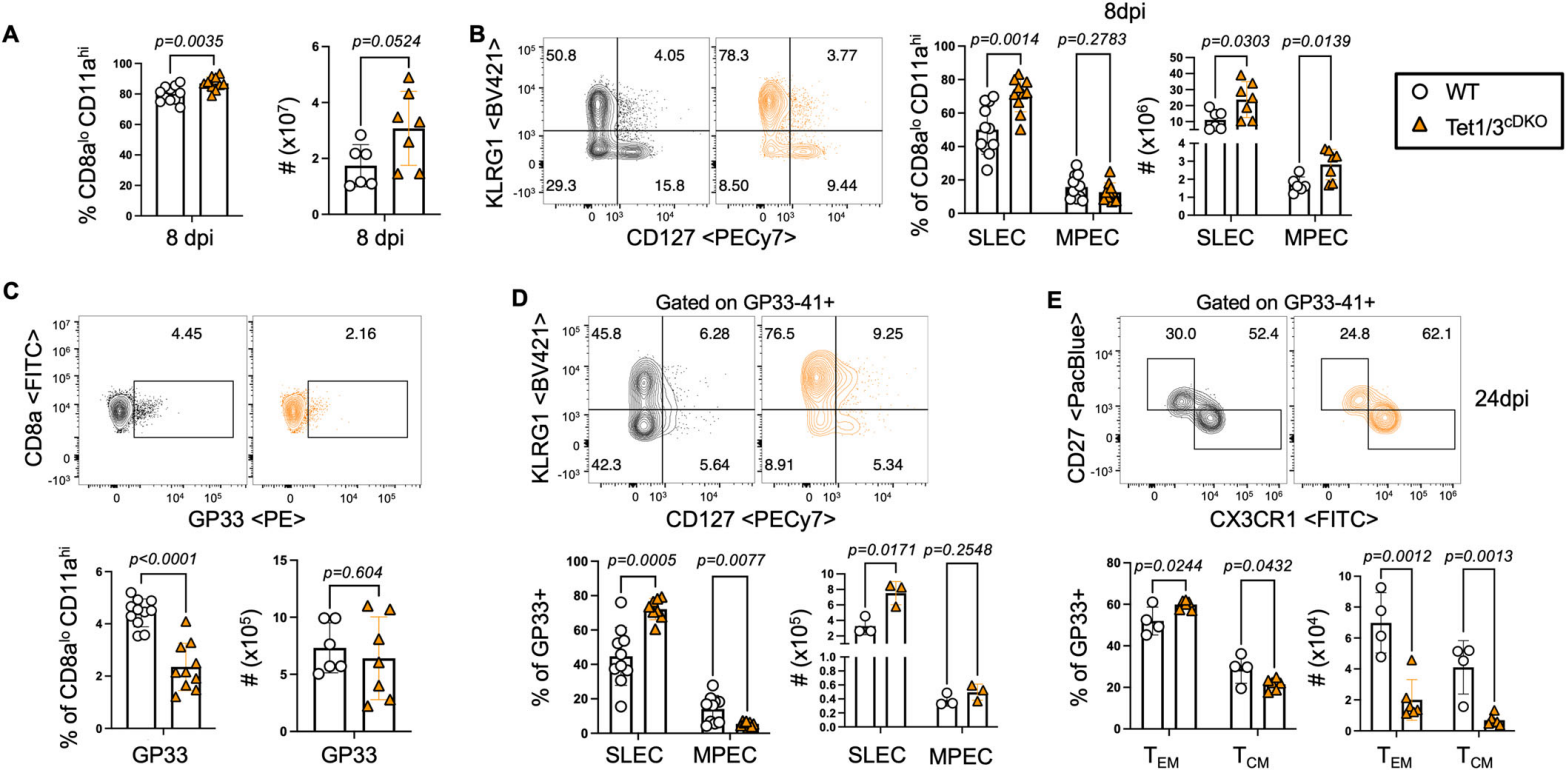
Tet1 and Tet3 deficiency results in increased SLECs and effector memory T-cell differentiation during acute LCMV infection. WT and Tet1/3^cDKO^ mice were infected with 2×10^5^ PFU of LCMV-Armstrong, and (**A**–**D**) blood was assessed 8dpi and (**E**) splenocytes were assessed 24dpi. (**A**) Percentage and number of antigen-experienced CD8 T cells. (WT = 11, Tet1/3^cDKO^ = 10 biological replicates) Data are mean ± SEM, unpaired *t* test. (**B**) Representative FACS plots comparing SLEC and MPEC populations by KLRG1 and CD127 expression and quantification of frequency and number of SLECs/MPECs, pregated on LiveTCRb+CD8a^lo^ CD11a^hi^ cells. (WT = 11, Tet1/3^cDKO^ = 10 biological replicates) Data are mean ± SEM, multiple unpaired *t* tests. (**C**) Representative FACS plots comparing GP33-tetramer+ CD8 T cells and quantification of frequency and number of tetramer-specific CD8 T cells, pregated on LiveTCRβ^+^CD8a^lo^ CD11a^hi^ cells. (WT = 11, Tet1/3^cDKO^ = 10 biological replicates) Data are mean ± SEM, unpaired *t* test. (**D**) Representative FACS plots and quantification of frequency and number of SLECs/MPECs among GP33-tetramer+ cells 8 dpi. (WT = 10, Tet1/3^cDKO^ = 8 biological replicates) Data are mean ± SEM, multiple unpaired *t* tests. (**E**) Representative FACS plots and quantification of frequency and number of T_EM_/T_CM_ cells among GP33-tetramer+ CD8 T cells 24dpi. (WT = 4, Tet1/3^cDKO^ = 6 biological replicates) Data are mean ± SEM, multiple unpaired *t* tests. Data information: *P* values are indicated in the figures and *P* < 0.05 was considered significant; paired male or female mice were used and no gender biases associated with genotypes were observed. (**A**–**E**) Experiments were replicated at least three times. Source data are available online for this figure.

Infection with the lymphocytic choriomeningitis virus (LCMV) Armstrong strain in C57BL/6 mice leads to an acute infection that is usually resolved within 8 days. At this juncture, the cellular numbers of antigen-specific CD8 T cells have peaked, and the cells have differentiated into effector cells (Wherry et al, 2003). Using Tet2^fl/fl^CD4Cre^Tg^ mice in which developing CD4 and CD8 T cells lack Tet2, it was previously shown that the loss of Tet2 promotes MPEC formation and T_CM_ CD8 T-cell differentiation in a cell-intrinsic manner following acute LCMV-Armstrong infection (Carty et al, 2018). To test the role of Tet1/3 in CD8 effector T-cell fates during acute LCMV-Armstrong infection, we challenged RorcCre^Tg^ Tet1/3^fl/fl^ mice (hereafter referred to as Tet1/3^cDKO^), in which double-positive thymocytes and their αβ CD4^+^ and CD8^+^ single positive progenies are deficient in Tet1 and Tet3 (Eberl and Littman, 2004; Issuree et al, 2018). Of note, Tet1/3^cDKO^ mice have comparable numbers of peripheral CD8 T cells compared to controls and quiescent CD4 and CD8 T-cell compartments (Fig. EV1A,B). Furthermore, the loss of Tet1/3 in CD8 T cells did not result in a compensatory increase in Tet2 mRNA expression (Fig. EV1C). Eight days post-infection (dpi) with LCMV-Armstrong, the proportions, and numbers of antigen-experienced Tet1/3^cDKO^ CD8 T cells, as assessed by low CD8a and positive CD11a expression, were modestly increased compared to WT littermate controls (Fig. 1A). The proportions and absolute numbers of SLECs among these cells, denoted by high KLRG1 and negative CD127 expression (Joshi et al, 2007), were significantly higher in Tet1/3^cDKO^ mice (Fig. 1B). Using MHC-I restricted epitope-specific D^b^-GP33-41, D^b^-GP276-286 and D^b^-NP396-404 tetramers, we found that the frequencies of tetramer-positive CD8 T cells in Tet1/3^cDKO^ mice were reduced, although the numerical numbers of these cells were comparable to WT controls (Figs. 1C and EV1D), plausibly due to a higher expansion of non-antigen-specific CD8 T cells in Tet1/3^cDKO^ mice. However, a significantly higher proportion of these epitope-specific T cells displayed a SLEC phenotype compared to WT controls (Figs. 1D and EV1E), suggesting that the propensity of Tet1/3^cDKO^ CD8 T cells to adopt a SLEC cell fate was not restricted to TCR specificities. Despite the increased frequency of SLECs in Tet1/3^cDKO^ mice, the viral copies of LCMV-Armstrong 5 dpi were not significantly different compared to WT mice (Fig. EV1F). T_EM_ and T_CM_ differentiation occur early during a primary T-cell response and persist in secondary lymphoid organs long after the infection is cleared (Chung et al, 2021). These populations can be identified through their differential expression of CD27 and CX3CR1, markers linked to functional maturation and T-cell trafficking in memory cells (Gerlach et al, 2016; Martin and Badovinac, 2018). While both GP33-41-specific T_EM_ and T_CM_ cells were numerically reduced in Tet1/3^cDKO^ mice due to the overall decrease in GP33-41-specific cells at 24 dpi (Figs. 1E and EV1G), we observed a markedly higher proportion of Db-GP33-41-specific T_EM_ cells compared to T_CM_ cells at both 24 and 8 dpi in Tet1/3^cDKO^ mice compared to WT controls (Figs. 1E and EV1G,H). Together, these data suggest that in contrast to the loss of Tet2, loss of Tet1/3 in developing T cells enhances SLECs and T_EM_ cell formation during acute LCMV infection, highlighting distinct roles for Tet1/2/3 during CD8 T-cell differentiation.

**Figure EV1:**

Tet1 and Tet3 deficiency results in increased SLECs and effector memory T-cell differentiation during acute LCMV infection. (**A**) Absolute numbers of mature CD8 + T cells from control Tet1/3fl/fl and Rorc(t)CreTgTet1/3fl/fl mice. (WT = 5, Tet1/3^cDKO^ = 8 biological replicates). Data are mean ± SEM, unpaired *t* test. (**B**) Representative FACS plots of CD62L and CD44 expression on CD8 T cells and frequency of naive CD8 T cells from indicated secondary lymphoid organs of control P14 Tet1/3fl/fl and P14-Rorc(t)CreTgTet1/3fl/fl mice, pregated on LiveTCRb+CD8a+ cells. (WT = 3, Tet1/3^cDKO^ = 3 biological replicates). Data are mean ± SEM, multiple unpaired *t* tests. (**C**) Relative expression of Tet2 normalized to HPRT in naive Tet1/3fl/fl and Rorc(t)CreTgTet1/3fl/fl CD8 T cells. (WT = 3, Tet1/3^cDKO^ = 2 biological replicates). (**D**) Representative FACS plots and quantification of frequency and number of GP276- and NP396-tetramer+ CD8 T cells. (WT = 3-4, Tet1/3^cDKO^ = 5 biological replicates) Data are mean ± SEM, multiple unpaired *t* tests. (**E**) Representative FACS plots and quantification of frequency and number of SLEC and MPECs among GP276-tetramer+ and NP396-tetramer+ CD8 cells. (WT = 3-4, Tet1/3^cDKO^ = 5 biological replicates) Data are mean ± SEM, multiple unpaired *t* tests. (**F**) RT-qPCR assessment of LCMV copy number (CN) per spleen 5dpi. (WT = 3, Tet1/3^cDKO^ = 3 biological replicates) Data are mean ± SEM, unpaired *t* test. (**G**) Absolute number of GP33-tetramer+ CD8 T cells 24dpi. (WT = 4, Tet1/3^cDKO^ = 6 biological replicates). Data are mean ± SEM, unpaired *t* test. (**H**) Representative FACS and quantification of frequency and number of TEM/TCM 8dpi. (WT = 10, Tet1/3^cDKO^ = 9 biological replicates). Data are mean ± SEM, multiple unpaired *t* tests. Data information: *P* values are indicated in the figures and *P* < 0.05 was considered significant; paired male or female mice were used and no gender biases associated with genotypes were observed. (**A**, **B**, **H**) Experiments were replicated at least three times. Source data are available online for this figure.

### Tet1/3 restrain the differentiation of SLECs and effector memory T cells in a cell-intrinsic manner

**Figure 2:**
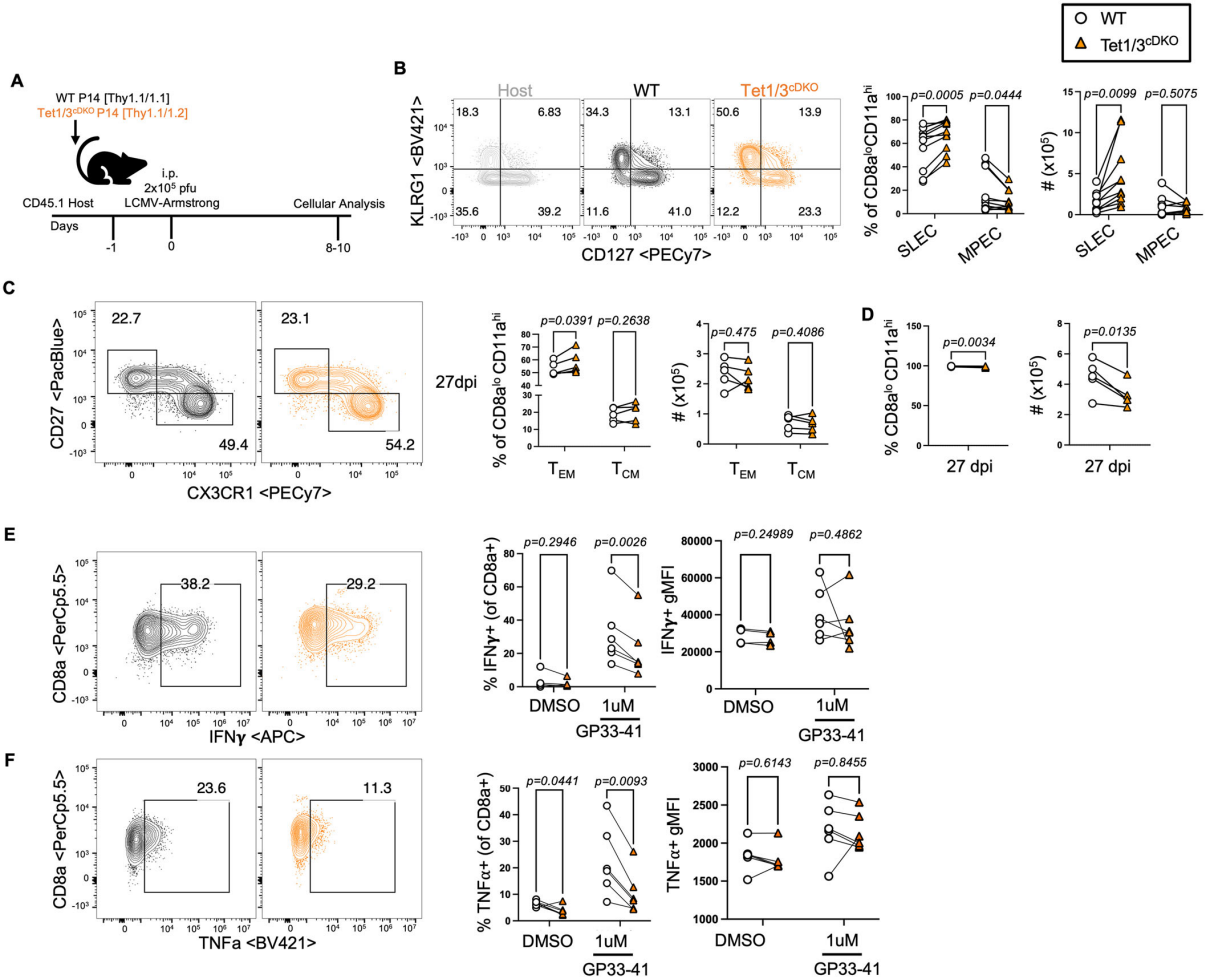
Tet1/3 restrain the differentiation of SLECs and effector memory T cells in a cell-intrinsic manner. In total, 5000 congenically labeled P14 WT (CD90.1/CD90.1) and 5000 P14 Tet1/3^cDKO^ (CD90.1/CD90.2) cells were adoptively co-transferred into naive CD45.1 hosts and infected with 2 × 10^5^ PFU of LCMV-Armstrong the following day and splenocytes were assessed 8-10dpi or 27dpi (**A**) Experimental set-up. (**B**) Representative FACS plots comparing SLEC/MPEC populations from the CD45.1 host, P14 WT, and Tet1/3^cDKO^ and quantification of frequency and number of SLECs/MPECs recovered 8dpi, pregated on LiveTCRb+CD8alo CD11ahi cells. (WT = 8, Tet1/3^cDKO^ = 8 biological replicates). Significance was determined by multiple paired t tests. (**C**) Representative FACS plots comparing T_EM_/T_CM_ populations from transferred P14 WT and Tet1/3^cDKO^ and quantification of frequency and number of T_EMs_/T_CMs_ recovered 27 dpi. Cells were pregated on LiveTCRb^+^CD8a^lo^ CD11a^hi^ cells. (WT = 5, Tet1/3^cDKO^ = 5 biological replicates) Significance was determined by multiple paired *t* tests. (**D**) Frequency and number of CD8a^lo^CD11a^hi^ among transferred P14 WT and Tet1/3^cDKO^ 27 dpi. (WT = 5, Tet1/3^cDKO^ = 5 biological replicates) Significance was determined by paired *t* test. (**E**) Representative flow plots and frequency of IFNγ+ and IFNγ gMFI among IFNγ + CD8^+^ T cells following ex vivo gp33-41 peptide restimulation of splenocytes in the presence of Brefeldin A and Monensin for 5 h. Splenocytes were isolated from mice 10 dpi (WT = 6, Tet1/3^cDKO^ = 6). Significance was determined by multiple paired *t* tests. (**F**) Representative flow plots and frequency of TNFα+ and TNFα gMFI among TNFα + CD8^+^ T cells following ex vivo gp33-41 peptide restimulation of splenocytes in the presence of Brefeldin A and Monensin for 5 h. Splenocytes were isolated from mice 10dpi (WT = 6, Tet1/3^cDKO^ = 6 biological replicates). Significance was determined by multiple paired *t* tests. Data information: *P* values are indicated in the figures and *P* < 0.05 was considered significant, paired male or female mice were used and no gender biases associated with genotypes were observed. (**B**–**D**) Experiments were replicated at least three times. Source data are available online for this figure.

To investigate whether the susceptibility of CD8 T cells to differentiate into SLECs and T_EM_ cells was due to cell-intrinsic requirements of Tet1/3, we transferred equal numbers of congenically- disparate WT or Tet1/3^cDKO^ P14 CD8^+^ T cells, which have a TCR restricted to the MHC-I-specific LCMV glycoprotein (GP) 33-41 peptide (Pircher et al, 1990), into WT hosts and infected them with LCMV-Armstrong a day later (Fig. 2A). LCMV infection led to comparable activation of WT or Tet1/3^cDKO^ P14 CD8^+^ T cells (Fig. EV2A), equivalent Ki67 expression in expanding antigen-experienced T cells (Fig. EV2B), and similar recovery of transferred WT and Tet1/3^cDKO^ T cells 5, 8, 10 and 14 dpi (Fig. EV2C), suggesting that Tet1/3 deficiency does not impact the activation or early proliferation of GP33-41-specific T cells. However, similar to polyclonal CD8 T cells from infected Tet1/3^cDKO^ mice, a higher frequency of Tet1/3^cDKO^ P14 cells differentiated into SLECs compared to WT P14 cells at multiple time points analyzed (Figs. 2B and EV2D). Similarly, Tet1/3^cDKO^ P14 cells preferentially differentiated into T_EM_ cells compared to WT controls at day8 and day27 dpi (Figs. EV2E and 2C). While the proportions of activated cells on day27 were similar, the total number of Tet1/3^cDKO^ P14 cells recovered was reduced (Fig. 2D), suggesting that the retention of this memory pool may be affected, although further longitudinal experiments are required. To ensure that memory populations detected by CD27 and CX3CR1 were bonafide T_EM_ and T_CM_ cells, we also assessed the expression of the transcription factor Tbet, which is expressed at higher levels in T_EM_ cells compared to T_CM_ cells (Joshi et al, 2007). Consistent with their transcriptional identities, we observed higher expression of Tbet in T_EM_ cells from both WT and Tet1/3^cDKO^ P14 T cells compared to the T_CM_ counterparts, and notably, Tbet expression was not affected by Tet1/3 deficiency (Fig. EV2F). Interestingly, upon ex vivo restimulation of splenocytes (isolated 10 dpi) with GP33-41 peptide, a lower proportion of Tet1/3^cDKO^ T cells produced IFNγ and TNF-α compared to WT controls (Fig. 2E,F), despite the increased frequency of SLECs and T_EM_-like cells among Tet1/3^cDKO^ T cells, suggesting effector differences in these populations. Taken together, these results suggest that Tet1/3 restrain the differentiation of SLECs and T_EM_ cells during acute LCMV infection in a cell-intrinsic manner.

**Figure EV2:**

Tet1/3 restrain the differentiation of SLECs and effector memory T cells in a cell-intrinsic manner. (**A**) Percent of CD8a^lo^CD11a^hi^ cells among transferred P14 CD8 T cells 8 dpi. (WT = 5, Tet1/3^cDKO^ = 5 biological replicates) Significance was determined by paired *t* test. (**B**) Ki67 MFI among transferred P14 CD8 T cells in the blood at 8 and 10 dpi. (WT = 5, Tet1/3^cDKO^ = 5 biological replicates) Significance was determined by multiple paired t tests. (**C**) Absolute numbers of transferred P14 CD8 T cells in the blood at indicated time points. (WT = 3, Tet1/3^cDKO^ = 3 biological replicates) Significance was determined by multiple paired *t* tests. (**D**) Representative FACS plots and quantification of frequency and number of SLECs recovered at indicated time points in the blood. (WT = 3, Tet1/3^cDKO^ = 3 biological replicates) Significance was determined by multiple paired *t* tests. (**E**) Representative FACS plots and quantification of frequency and number of TEM/TCM cells recovered 8 dpi. (WT = 8, Tet1/3^cDKO^ = 8 biological replicates) Significance was determined by multiple paired *t* tests. (**F**) Representative histograms and quantification of Tbet expression in TEM/TCM populations. (WT = 5, Tet1/3^cDKO^ = 5 biological replicates) Significance was determined by multiple paired *t* tests. Data information: *P* values are indicated in the figures and *P* < 0.05 was considered significant; paired male or female mice were used and no gender biases associated with genotypes were observed. (**A**–**D**) Experiments were replicated at least three times. Source data are available online for this figure.

### Tet1/3 restrict SLEC/T_EM_ CD8 T-cell differentiation by controlling TCR-dependent epigenetic circuits

**Figure 3:**
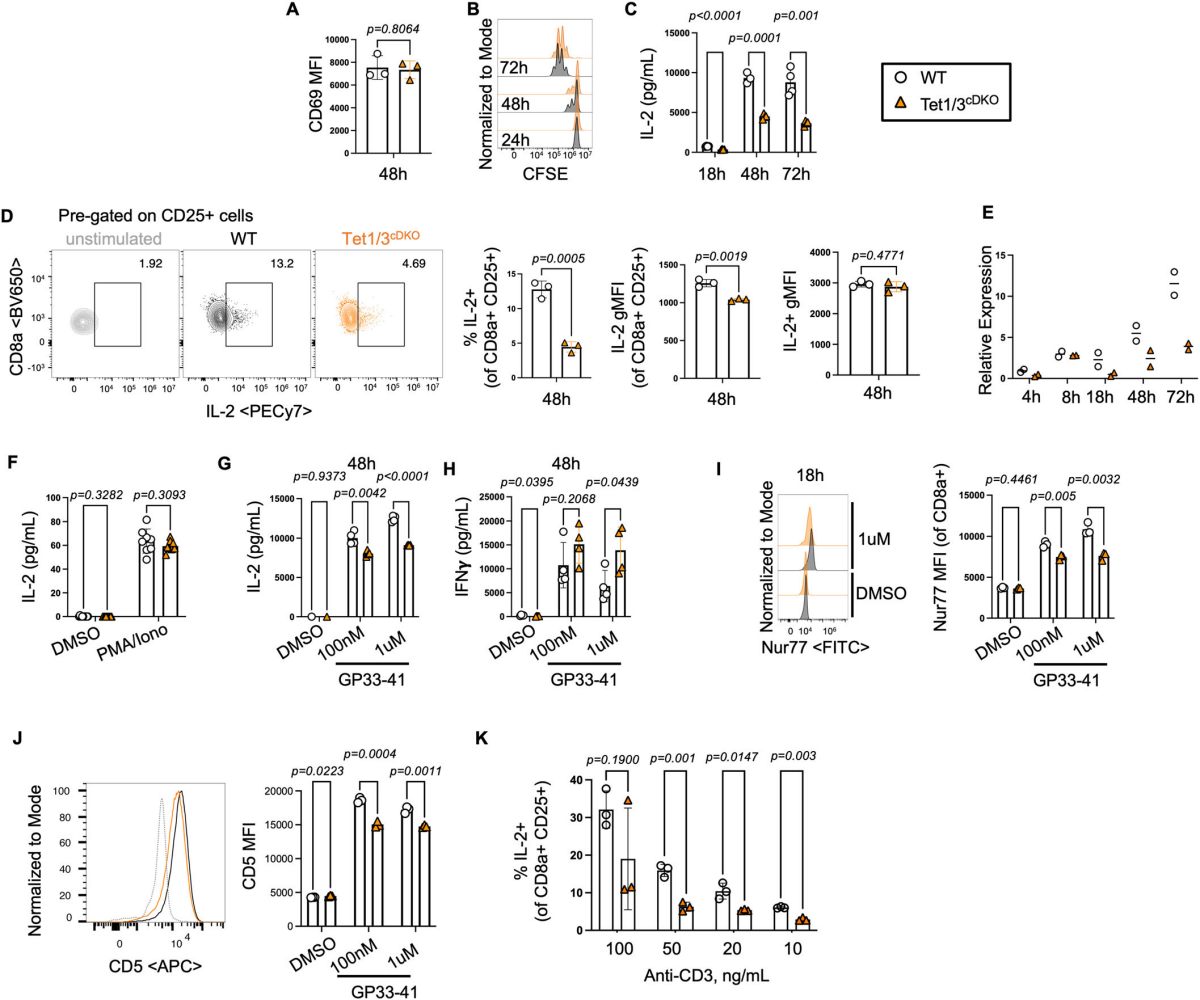
Tet1/3 restrict SLEC/T_EM_ CD8 T-cell differentiation by controlling TCR-dependent epigenetic circuits. (**A**–**F**) FACS-sorted WT and Tet1/3^cDKO^ CD8 T cells were stimulated for indicated time points in vitro using plate-bound anti-CD3 and anti-CD28 (**G**–**J**) P14 WT and Tet1/3^cDKO^ splenocytes from naive mice were stimulated in vitro with gp33-41 peptide and anti-CD28 for indicated time points. (**A**) CD69 MFI on CD8 T cells. (WT = 3, Tet1/3^cDKO^ = 3 biological replicates). Data are mean ± SEM, unpaired *t* test. (**B**) Representative histogram showing CD8 T-cell proliferation at indicated time points by CFSE dilution. (**C**) Quantification of IL-2 levels in the supernatants of activated CD8 T cells by ELISA. (WT = 3-4, Tet1/3^cDKO^ = 4 biological replicates). Data are mean ± SEM, multiple unpaired *t* tests. (**D**) Representative FACS plots and quantification of IL-2+ cells, gMFI of IL-2 in bulk CD8 + CD25 + T cells, gMFI of IL-2 among IL-2+ cells, 48-h post activation. Cells were treated with Brefeldin A and Monensin for the last 5 h of stimulation. (WT = 3, Tet1/3^cDKO^ = 3 biological replicates). Data are mean ± SEM, unpaired *t* test. (**E**) Relative expression of *Il2* normalized to HPRT, by RT-qPCR. (WT = 2, Tet1/3^cDKO^ = 2 biological replicates). (**F**) Quantification of IL-2 by ELISA in the supernatants of naive CD8 T-cell stimulated with PMA/Ionomycin for 5 h (WT = 8, Tet1/3^cDKO^ = 8 biological replicates). Data are mean ± SEM, multiple unpaired *t* tests. (**G**) Quantification of IL-2 by ELISA from supernatants (WT = 4, Tet1/3^cDKO^ = 4 biological replicates). Data are mean ± SEM, multiple unpaired *t* tests. (**H**) Quantification of IFNγ by ELISA from supernatants (WT = 4, Tet1/3^cDKO^ = 4 biological replicates). Data are mean ± SEM, multiple unpaired *t* tests. (**I**) Representative histogram and quantification of Nur77 MFI among LiveTCRb+CD8a + CD69+ cells. (WT = 3, Tet1/3^cDKO^ = 3 biological replicates). Data are mean ± SEM, multiple unpaired *t* tests. (**J**) Representative histogram and quantification of CD5 MFI among LiveTCRb+CD8a + CD69+ cells. (WT = 3, Tet1/3^cDKO^ = 3 biological replicates). Data are mean ± SEM, multiple unpaired *t* tests. Dotted line on the histogram shows CD5 expression in unstimulated T cells. (**K**) Frequency of IL-2+ cells among CD8 + CD25 + T cells upon 48 h stimulation with varying doses of anti-CD3 and a constant dose of 500 ng/mL CD28. (WT = 3, Tet1/3^cDKO^ = 3 biological replicates). Data are mean ± SEM, multiple unpaired *t* tests. Data information: *P* values are indicated in the figures and *P* < 0.05 was considered significant, paired male or female mice were used, and no gender biases associated with genotypes were observed. (**B**–**D**) Experiments were replicated at least two times. Source data are available online for this figure.

We previously found that Tet1/3 were required for the differentiation of thymic regulatory T cells by regulating both the generation of Treg cell progenitors and TCR-dependent IL-2 production in CD4^+^ thymocytes (Teghanemt et al, 2023). To investigate the cell-intrinsic mechanisms that promote SLEC and T_EM_ cell differentiation in the absence of Tet1/3, we examined whether TCR-dependent activation of CD8 T cells and subsequent responses were modified in cells lacking Tet1/3. Naive CD8 T cells from Tet1/3^cDKO^ mice were FACS-sorted and stimulated in vitro with anti-CD3/CD28 over a time course. Both WT and Tet1/3^cDKO^ CD8 T cells expressed similar levels of CD69 (Fig. 3A), an early gene upregulated downstream of TCR stimulation and divided comparably over 72 h (Fig. 3B). However, Il-2 production from Tet1/3cDKO cells was profoundly reduced as assessed by both ELISA and correlated with a significantly lower proportion of IL-2 producing cells as assessed by flow cytometry, although the amount of IL-2 secreted in by IL-2+ Tet1/3cDKO cells was comparable to WT cells (Fig. 3C,D). The reduced frequency of IL-2-producing cells correlated with decreased IL-2 mRNA transcripts in bulk CD8 T cells, upon TCR activation (Fig. 3E). However, short-term stimulation of naive CD8 T cells with PMA and ionomycin, which trigger signaling pathways downstream of the TCR but circumvent the TCR itself (Chatila et al, 1989), led to equivalent IL-2 levels (Fig. 3F), suggesting that the defect in IL-2 production by Tet1/3^cDKO^ cells was dependent on TCR-mediated stimulation. In further support of this notion, stimulation of P14 Tet1/3^cDKO^ CD8 T cells with GP33-41 also led to reduced IL-2 production over time compared to controls (Figs. 3G and EV3A), although they expressed similar CD69 expression and divided equally as WT controls (Fig. EV3B,C). Notably, IFN-γ production was not impaired and showed a trending increase in Tet1/3^cDKO^ T cells (Fig. 3H), suggesting that Tet1/3 deficiency in CD8 T cells regulates selective effector responses downstream of TCR activation. Interestingly, Nr4a1 (also known as Nur77), an early response gene that is rapidly upregulated following TCR signaling (Ashouri and Weiss, 2017; Bending et al, 2018; Moran et al, 2011; Zikherman et al, 2012) and used as a proxy for TCR signaling strength, was significantly reduced in peptide-stimulated Tet1/3^cDKO^ P14 T cells (Fig. 3I). This reduction in TCR signaling strength was also captured by examining CD5 expression levels, which were significantly reduced in Tet1/3^cDKO^ P14 cells (Fig. 3J). However, increasing TCR signaling strength by increasing peptide antigen load or increasing Cd3e stimulation did not overcome the reduced ability of Tet1/3cDKO cells to produce IL-2 (Fig. 3G,K). Upon pMHC engagement, the TCR triggers the activation of Erk, which is activated within minutes of antigen encounter and, over these short timescales, appears to function in an all-or-nothing, “digital” manner and exhibit similar signaling levels regardless of pMHC affinity or dose (Altan-Bonnet and Germain, 2005; Gallagher et al, 2021). Importantly, IL-2 expression in T cells is dependent on the binding of the AP-1 family of transcription factors upon TCR activation, which requires ERK pathway activation (Janulis et al, 1999; Samelson, 2002; Walters et al, 2013). Surprisingly, we did not observe impairments in ERK phosphorylation in Tet1/3^cDKO^ T cells upon TCR activation (Fig. EV3D), suggesting that epigenetic circuits that integrate upstream signals are likely impaired in Tet1/3cDKO cells. We previously showed that the ability of CD4 T cells to make IL-2 was selectively dependent on Tet3 (Teghanemt et al, 2023). As expected, we found that IL-2 production in Tet3-deficient CD8 T cells was profoundly impaired following TCR activation. To our surprise, however, Tet3 cKO mice did not show a significant increase in SLEC or T_EM_ differentiation 8dpi with LCMV, and the proportion of GP33-specific tetramer cells was not reduced, although we did not perform a refined kinetic assessment of SLEC differentiation. As autocrine IL-2 is not required for effector CD8 T-cell differentiation during primary infection (Toumi et al, 2022) and considering the lack of SLEC/T_EM_ phenotypes in Tet3 cKO mice, we conclude that Tet3 is not sufficient to restrict SLEC/T_EM_ CD8 T-cell differentiation and that Tet1 or the combined actions of Tet1 and Tet3 are likely required for this process.

**Figure EV3:**

Tet1/3 restrict SLEC/T_EM_ CD8 T-cell differentiation by controlling TCR-dependent epigenetic circuits. (**A**–**C**) Naive P14 WT and Tet1/3^cDKO^ splenocytes were stimulated in vitro with gp33-41 peptide and anti-CD28 for indicated time points. (**D**) FACS-Sorted naive WT and Tet1/3^cDKO^ T cells were stimulated in vitro via anti-CD3 and anti-CD28 for indicated time points. (**A**) Representative FACS plots and quantification of frequency of IL-2+ cells among CD69+ cells, gMFI of IL-2 among CD69+ cells, gMFI of IL-2 among IL-2^+^ cells, 48-h post activation. Cells were treated with Brefeldin A and Monensin for the last 5 h of stimulation (WT = 3, Tet1/3^cDKO^ = 3 biological replicates). Data are mean ± SEM, multiple unpaired *t* tests. (**B**) CD69 MFI on CD8 T cells 48-h post activation (WT = 3, Tet1/3^cDKO^ = 3 biological replicates). Data are mean ± SEM, multiple unpaired *t* test. (**C**) Representative histogram showing CD8 T-cell proliferation by CFSE dilution. (**D**) Representative immunoblot and quantification of phospho-ERK1/2. Blots were quantified using ImageJ and normalized to show quantification of three independent experiments. Data are mean ± SEM, multiple unpaired *t* tests. (**E**) Quantification of IL-2 levels in the supernatants of activated CD8 T cells by ELISA. (WT = 4, Tet3^cKO^ = 4 biological replicates). Data are mean ± SEM, multiple unpaired *t* tests. (**F**) Percentage and number of antigen-experienced CD8 T cells 8dpi. (WT = 4, Tet3^cKO^ = 4 biological replicates) Data are mean ± SEM, unpaired *t* test. (**G**) Representative FACS plots and quantification of frequency and number of GP33-tetramer+ CD8 T cells. Cells were pregated on LiveTCRβ^+^CD8a^lo^ CD11a^hi^ cells. (WT = 4, Tet3^cKO^ = 4 biological replicates) Data are mean ± SEM, unpaired *t* test. (**H**) Representative FACS plots and quantification of frequency and number of SLECs/MPECs among GP33-tetramer+ cells 8 dpi. (WT = 4, Tet3^cKO^ = 4 biological replicates) Data are mean ± SEM, multiple unpaired *t* tests. (**I**) Quantification of frequency of SLECs/MPECs among activated CD8 T cells 8 dpi. Data is a summary of 2 independent experiments (WT = 8, Tet3^cKO^ = 10 biological replicates) Data are mean ± SEM, multiple unpaired *t* tests. Data information: *P* values are indicated in the figures and *P* < 0.05 was considered significant; paired male or female mice were used and no gender biases associated with genotypes were observed. (**A**) Experiments were replicated three times. (**B**–**D**) Experiments were replicated twice. Source data are available online for this figure.

### Loss of Tet1/3 impairs chromatin accessibility at regulatory regions in TCR-responsive genes

**Figure 4:**
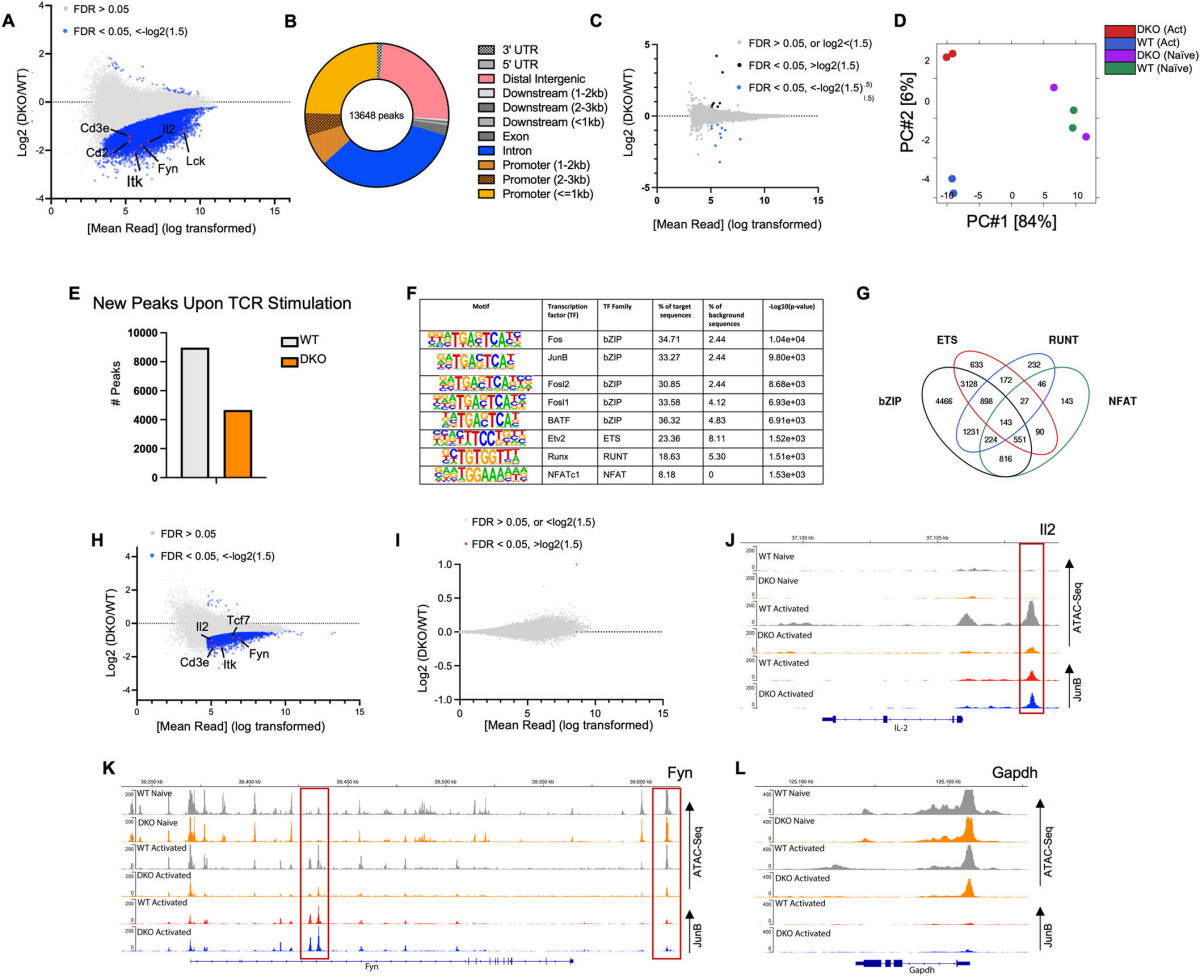
Loss of Tet1/3 impairs chromatin accessibility at regulatory regions in TCR-response genes. (**A**) Scatter plot depicting regions with differences in chromatin accessibility between activated Tet1/3^cDKO^ and WT CD8 T cells. T cells were activated for 18 h with anti‐CD3/CD28, *n* = 2 biological replicates/genotype. (**B**) Pie Chart depicting the proportions of differentially accessible peaks between activated Tet1/3^cDKO^ and WT CD8 T cells within distinct genomic regions. (**C**) Scatter plot depicting regions with differences in chromatin accessibility in ex vivo FACs-sorted naive Tet1/3^cDKO^ and WT CD8 T cells, *n* = 2 biological replicates/genotype. (**D**) PCA plot showing distinct clusters of samples based on genotype and treatment. (**E**) Bar graph showing the number of de novo accessible peaks that are open following TCR activation in WT T cells and the number of peaks present in Tet1/3^cDKO^ counterparts (FDR < 0.05). (**F**) Enriched DNA motifs in differentially accessible regions between activated Tet1/3^cDKO^ and WT CD8 T cells. The table shows the percentage of regions with motifs identified by HOMER analysis and selected with an adjusted *P* value ≤ 10^−5^ and target/background >2. (**G**) Overlap of differentially accessible regions containing bZIP, ETS, Runt or NFAT motifs in in vitro-activated Tet1/3^cDKO^ and WT CD8 T cells. (**H**) Scatter plot depicting regions with differences in chromatin accessibility between P14 Tet1/3^cDKO^ and WT T cells isolated 5dpi with LCMV-Armstrong, *n* = 3 biological replicates/group. (**I**) Scatter plot depicting differential JunB peaks identified by Cut and Tag in Tet1/3^cDKO^ and WT T cells 18 h after TCR activation, *n* = 3 biological replicates/group. (**J**–**L**) Integrated genome browser view (IGV) shots of the *Il-2*, *Fyn,* and *Gapdh* genes. Tracks show the reference gene, ATAC-Seq peaks (WT in gray, Tet1/3^cDKO^ in orange), and JunB Cut and Tag peaks (WT in red and Tet1/3^cDKO^ in blue) in naive or activated cells.

**Figure EV4:**
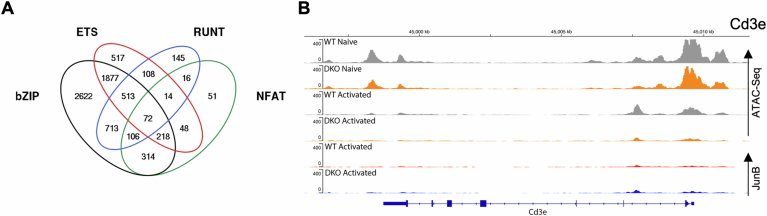
Loss of Tet1 and Tet3 impairs chromatin accessibility at regulatory regions in TCR-response genes. (**A**) Overlap of differentially accessible peaks containing bZIP, ETS, Runt or NFAT in P14 Tet1/3^cDKO^ and WT CD8 T cells isolated 5dpi with LCMV-Armstrong (**B**) Integrated genome browser view (IGV) shots of the Cd3e gene. Tracks show the reference gene, ATAC-Seq peaks (WT in gray, Tet1/3^cDKO^ in orange) and JunB Cut and Run peaks (WT in red and Tet1/3^cDKO^ in blue) in naive or activated cells.

To interrogate how Tet1/3 regulate selective TCR-dependent transcriptional programs epigenetically, we assessed the chromatin accessibility status of genes by ATAC-Seq (Buenrostro et al, 2015). As previously reported in human and murine T cells (Bevington et al, 2016; Wang et al, 2018; Yukawa et al, 2020), early TCR stimulation led to a marked change in chromatin remodeling in a large proportion of genes, with 38,130 differentially accessible peaks detected between activated and naive WT T cells (Fold change >1.5, FDR < 0.05, Dataset EV1). However, the number of accessible chromatin regions in activated Tet1/3^cDKO^ cells was profoundly reduced compared to WT controls (Fig. 4A). The majority of these differential chromatin regions were situated in the intragenic and intergenic regions of genes (Fig. 4B) and were associated with genes involved in regulation of TCR-associated pathways, including *Fyn, Itk, Lck, Cd3e*, and *Il-2* (Fig. 4A). In contrast, Tet1/3 deficiency had a very modest impact on the chromatin landscape of genes in naive CD8 T cells (Fig. 4C,D), suggesting that Tet1/3 were mainly required for promoting accessibility in TCR-responsive regulatory regions. In agreement with this, we found that TCR stimulation led to de novo opening of 8980 chromatin regions in WT cells, and 52% of these regions (4669) remained closed in Tet1/3^cDKO^ T cells (Fig. 4E; Dataset EV2). Homer analysis revealed that motifs belonging to the bZIP, ETS, NFAT, and RUNT family of transcription factors (TF) were significantly enriched in differentially accessible regions between Tet1/3^cDKO^ and WT cells (Fig. 4F), with approx. 94% of all differential peaks containing motifs belonging to at least one of these four families. Interestingly, bZIP TF motifs were greatly co-enriched with ETS TF motifs, although a significant fraction of differential peaks only contained bZIP TF motifs (Fig. 4G). ATAC-Seq profiling in P14 T cells isolated from mice 5 dpi with LCMV-Armstrong also revealed significantly reduced chromatin accessibility in Tet1/3^cDKO^ CD8 T cells compared to WT controls. These differences occurred in TCR-associated genes as well as in TF gene loci previously linked to effector and memory T-cell differentiation, such as *Tcf7 and Runx3* (Fig. 4H, Dataset EV3) (Pais Ferreira et al, 2020; Schauder et al, 2021; Wang et al, 2018; Zhou et al, 2010). Mirroring results derived from in vitro-activated T cells, differentially accessible regions in P14 T cells were also highly enriched in motifs belonging predominantly to the bZIP, ETS, NFAT and RUNT family of TFs (Fig. EV4A). The bZIP family of TFs include the AP-1 family and Bach family members which form heterodimeric complexes at palindromic 12-O-Tetradecanoylphorbol-13-acetate response elements (TRE; 5’-TGA(C/G)TCA-3’) (Glover and Harrison, 1995; Turner and Tjian, 1989). In contrast to AP-1 TFs which act as transcriptional activators, Bach proteins act as transcriptional repressors and together these TFs play a central role in coordinating TCR-driven effector programs during infection (Oyake et al, 1996; Richer et al, 2016; Roychoudhuri et al, 2016). As JunB is a critical AP-1 member recruited downstream of TCR stimulation and modulates IL-2 production (Katagiri et al, 2019; Yukawa et al, 2020), we assessed whether Tet1/3 deficiency impaired JunB binding. Genome-wide JunB profiling by Cut and Tag (Kaya-Okur et al, 2019) revealed that 27,601 peaks of the 33,941 detected peaks (FDR < 0.05) had an AP-1 motif, underscoring the reliability of the assay (Dataset EV4). However, JunB binding was largely unaffected in Tet1/3^cDKO^ cells (Fig. 4I), including at a putative upstream IL-2 enhancer element and within differentially accessible sites in the *Fyn* locus (Fig. 4J,K). Of note, JunB binding was undetectable within the *Gapdh* and *Cd3e* genes (Figs. 4L and EV4B). Altogether, these findings suggest that Tet1/3-mediated DNA demethylation are required for chromatin accessibility at regulatory regions of key genes downstream of TCR activation independently of JunB binding.

### CD8 T cells undergo Tet1/3-dependent DNA demethylation during thymic development

**Figure 5:**
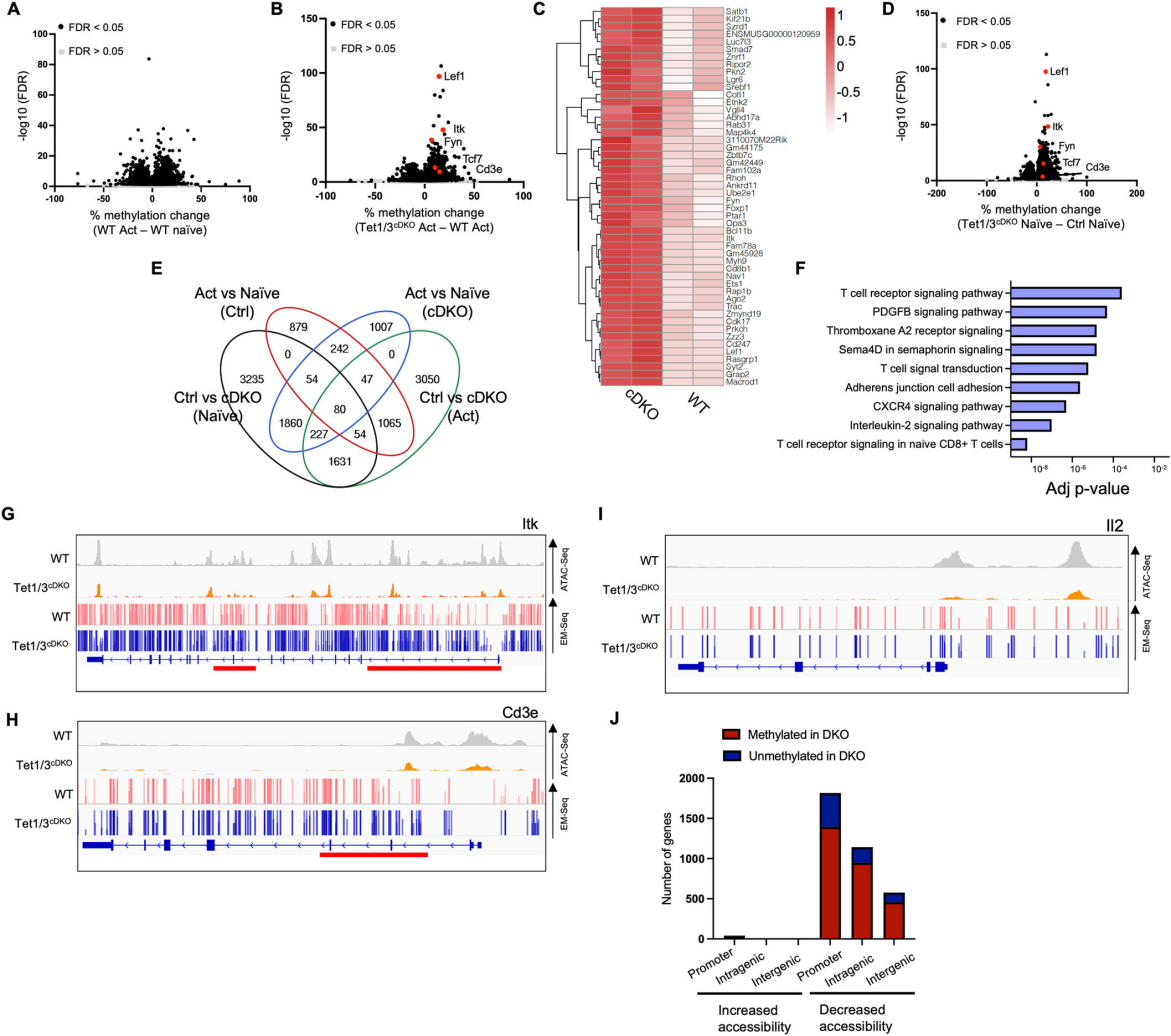
CD8 T cells undergo Tet1/3-dependent DNA demethylation during thymic development. (**A**) Volcano plot depicting % methylation change in gene loci in WT CD8 T cells upon TCR activation for 18 h, *n* = 2 biological replicates/group. (**B**) Volcano plot depicting % methylation change in gene loci between activated Tet1/3^cDKO^ and WT CD8 T cells *n* = 2 biological replicates/group. (**C**) Heatmap representing the top 50 differentially methylated gene loci between activated Tet1/3^cDKO^ and WT CD8 T cells. (**D**) Volcano plot depicting % methylation change in gene loci between naive Tet1/3^cDKO^ and WT CD8 T cells, *n* = 2 biological replicates/group. (**E**) Overlap of differentially methylated gene loci in various treatment and genotype comparisons indicated. (**F**) Gene enrichment analysis of differentially methylated gene loci between naive Tet1/3^cDKO^ and WT CD8 T cells. Adjusted *P* value was calculated using the Benjamini–Hochberg method. and *P* < 0.05 was considered significant. (**G**–**I**) Integrated genome browser view (IGV) shots of the Itk, IL-2, and Cd3e genes. Tracks show the reference gene, ATAC-Seq peaks (WT in gray, Tet1/3^cDKO^ in orange), and EM-Seq methylation peaks (WT in red and Tet1/3^cDKO^ in blue) in activated T cells. (**J**) Bar graph showing the number of gene loci with increased or decreased chromatin accessibility in the promoter, intergenic, or intragenic regions of Tet1/3^cDKO^ cells and having increased or reduced DNA methylation within the gene body and promoter regions (FDR < 0.1). Source data are available online for this figure.

**Figure EV5:**
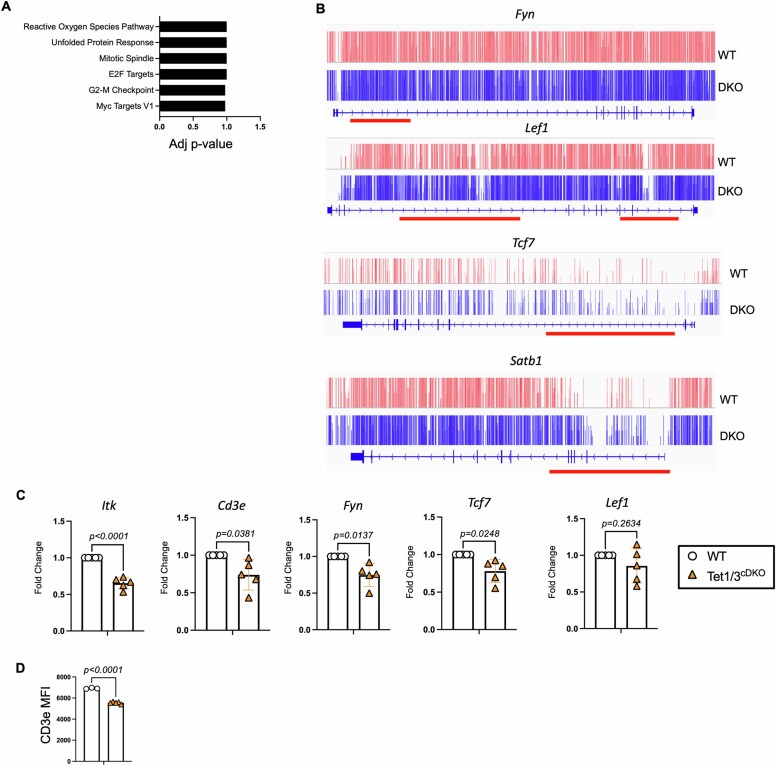
CD8 T cells undergo Tet/3-dependent DNA demethylation during thymic development. (**A**) Gene enrichment analysis of differentially methylated gene loci between WT activated and naive CD8 T cells. Adjusted *P* value was calculated using the Benjamini–Hochberg method. (**B**) Integrated genome browser view (IGV) shots of the Fyn, Lef1, Tcf7 and Satb1 gene loci. Tracks show the reference gene, and EM-Seq methylation peaks (WT in red and Tet1/3^cDKO^ in blue) in activated CD8 T cells. Red bar indicates regions with differentially methylated CpGs. (**C**) Fold change of Itk, Cd3e, Fyn, Tcf7 and Lef1 expression in activated CD8 T cells by RT-qPCR. Data is normalized to HPRT. (WT = 5, Tet1/3^cDKO^ = 5 biological replicates). Cells were activated in vitro for 48 h with anti-CD3/CD28. Data are mean ± SEM, unpaired *t* test. (**D**) CD3e MFI 48 h post activation with anti-CD3/CD28. (WT = 3, Tet1/3^cDKO^ = 5 biological replicates). Data are mean ± SEM, unpaired t test. Data information: *P* values are indicated in the figures and *P* < 0.05 was considered significant; paired male or female mice were used and no gender biases associated with genotypes were observed. (**A**) Experiments were replicated three times. (**C**, **D**) Experiments were replicated at least three times. Source data are available online for this figure.

Following 4–8 days of LCMV infection, a significant number of genes become hypomethylated in CD8 T cells, particularly in effector gene loci (Scharer et al, 2013; Youngblood et al, 2017). The degree to which this is due to active demethylation rather than passive loss of methylation during T-cell replication and whether early changes in DNA demethylation dictate cell fate programs remain unclear. We therefore investigated the contribution of Tet1/3-dependent demethylation during early TCR stimulation by examining genome-wide DNA methylation by enzymatic methyl sequencing (EM-Seq) (Vaisvila et al, 2021). Like bisulfite sequencing, EM-Seq does not distinguish between 5mC and 5hmC, reading both as a methylated cytosine residue and are thus referred to as 5mC/5hmC to indicate detection of either. CpG sites with at least 3× coverage were retained in the downstream EM-Seq analysis and covered an average of 3.6 × 10^6^ individual CpGs. 16 h post-anti-CD3/CD28 mediated TCR stimulation, when T cells had not yet undergone cell division (Fig. 3B), we identified 7758 differentially methylated gene loci between WT naive and activated CD8 T cells. Of these, 2421 gene loci showed reduced 5mC/5hmC in activated CD8 T cells compared to naive counterparts (Fig. 5A; Dataset EV5). However, they were not enriched in the regulation of specific pathways but broadly associated with the regulation of cellular processes such as mitosis (Fig. EV5A). 423 of these loci (12%) also showed reduced 5mC/5hmC when Tet1/3^cDKO^ CD8 T cells were activated, suggesting that these genes lost methylation in a Tet1/3-independent manner following TCR activation. Remarkably, 8484 loci had differential methylation between activated WT and Tet1/3^cDKO^ T cells, of which 6154 loci showed significantly reduced 5mC/5hmC in WT cells compared to Tet1/3^cDKO^ (Fig. 5B). The top 50 gene loci included *Itk* and *Fyn* (Fig. 5B,C) which are important mediators of antigen receptor signaling in T cells (Andreotti et al, 2010; Palacios and Weiss, 2004). Other gene loci with significantly reduced methylation in WT cells compared to Tet1/3^cDKO^ counterparts included *Itk, Cd3e*, *Tcf7, Lef1*, and *Satb1* (Fig. EV5B). Increased 5mC/5hmC marks in these cells correlated with a significant reduction in mRNA transcripts of these genes in activated Tet1/3^cDKO^ CD8 T cells compared to WT controls (Fig. EV5C), and we observed lower Cd3e protein expression in Tet1/3^cDKO^ CD8 T cells (Fig. EV5D). Given that a significantly lower number of genes undergo a loss in methylation upon T-cell activation in WT cells, we tested whether this result was due to pre-existing methylation present in naive Tet1/3^cDKO^ cells. Indeed, we identified 7141 loci that have higher 5mC/5hmC in Tet1/3^cDKO^ naive T cells compared to WT naive controls (Fig. 5D,E), suggesting that Tet1/3 are required for shaping the DNA demethylation landscape in mature CD8 T cells. These results are concordant with previous reports that T cells experience a dramatic increase in 5hmC marks in the bodies of genes as they undergo lineage differentiation into CD4 and CD8 T cells from DP precursors in the thymus (Rodriguez et al, 2015; Sellars et al, 2015; Tsagaratou et al, 2014). Strikingly, gene enrichment analysis revealed that genes predominantly enriched in regulatory pathways associated with IL-2 signaling and TCR signaling, such as *Itk*, *Cd3e, Fyn, Lck* were dependent on the activities of Tet1/3 (Figs. 5F–H and EV5C). Consistent with our previous findings in CD4 T cells (Teghanemt et al, 2023), we did not identify significant differences in methylation proximal or upstream to the IL-2 gene promoter between WT and Tet1/3^cDKO^ cells (Fig. 5I), indicating that the defect in IL-2 production in Tet1/3 CD8 T cells was likely due to expression defects in modulators of the TCR signaling pathway. We next asked whether there was a correlation between increased 5mC/5hmC marks present *in cis* with changes in chromatin regions in regulatory regions of gene loci in Tet1/3cDKO cells. Indeed, a large number of genes with increased 5mC/5hmC marks present in the gene body+3 kb upstream of the promoter had decreased chromatin accessibility in their promoter, intragenic, and intergenic regions (Fig. 5J), in concordance with a numerous body of literature showing the negative correlation between gene body DNA methylation and chromatin accessibility (Greenberg and Bourc’his, 2019). Taken together, these data support the notion that Tet1/3-mediated demethylation is critical during thymic development to license the chromatin accessibility landscape of genes following TCR activation.

### Tet1/3 are dispensable in peripheral CD8 T cells for effector and memory differentiation

**Figure 6:**
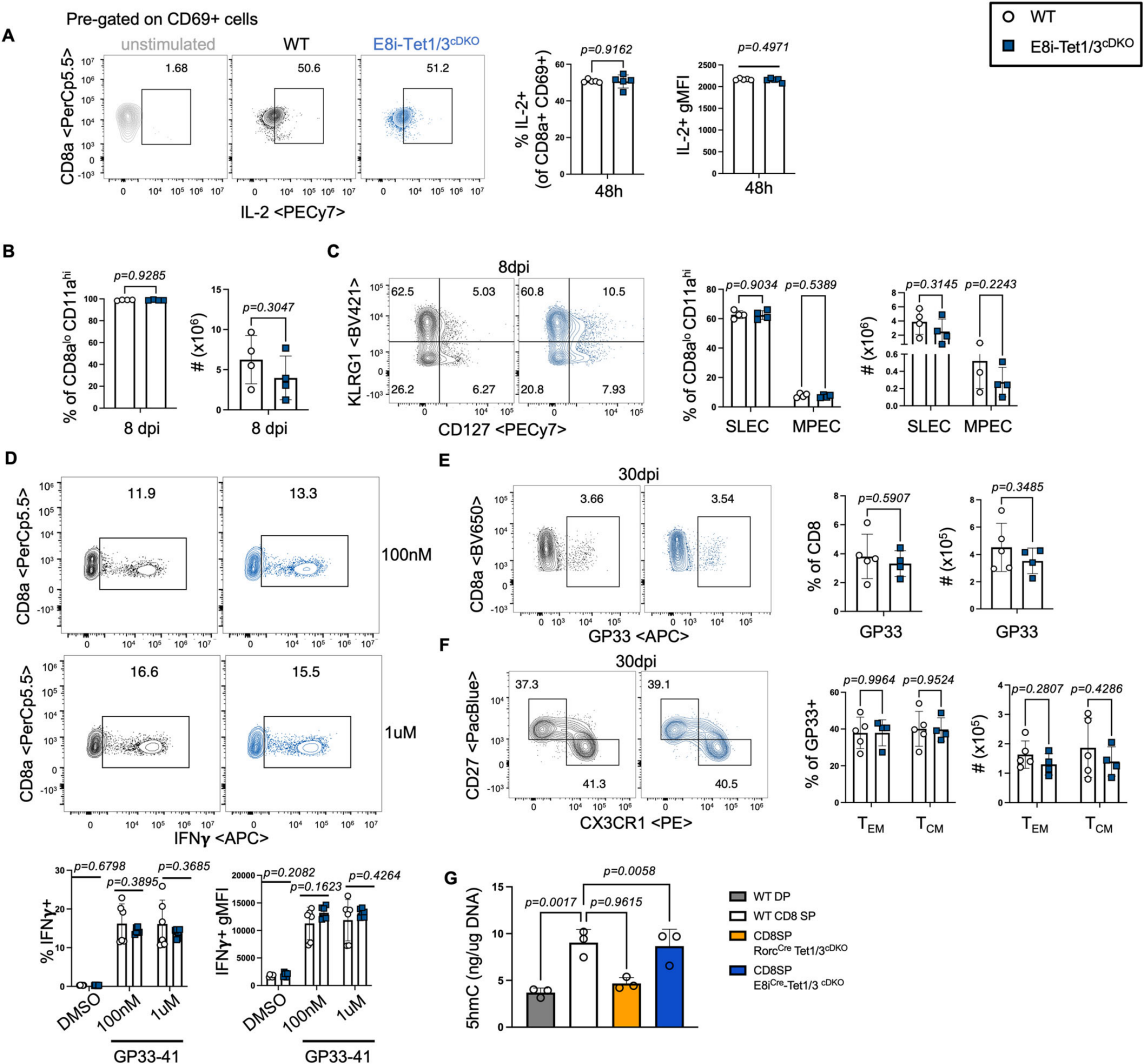
Tet1/3 are dispensable in peripheral CD8 T cells for effector and memory cell differentiation. (**A**) Representative FACS plots and quantification of frequency of IL-2+ and gMFI of IL-2+ cells, 48 h post activation of FACS-sorted naive T cells with anti-CD3 and anti-CD28. Cells were treated with Brefeldin A and Monensin for the last 5 h of stimulation. (WT = 5, Tet1/3^cDKO^ = 5 biological replicates). Data are mean ± SEM, unpaired *t* test. (**B**–**F**) WT and E8iCretgTet1/3fl/fl mice were infected with 2 × 10^5^ PFU of LCMV-Armstrong. (**B**) Percentage and number of antigen-experienced CD8 T cells 8dpi. (WT = 4, Tet1/3^cDKO^ = 4 biological replicates) Data are mean ± SEM, unpaired *t* test. (**C**) Representative FACS plots and quantification of frequency and number of SLECs/MPECs from the spleen 8dpi (WT = 4, Tet1/3^cDKO^ = 4 biological replicates) Data are mean ± SEM, multiple unpaired *t* tests. (**D**) Frequency of IFNγ-producing and IFNγ gMFI among IFNγ + CD8 T cells following gp33-41 peptide restimulation of splenocytes, isolated from mice 8dpi. (WT = 6, Tet1/3^cDKO^ = 6 biological replicates). Data are mean ± SEM, multiple unpaired *t* tests. (**E**) Representative FACS plots and quantification of frequency and number of GP33-tetramer+ CD8 T cells in the spleen 30 dpi. (WT = 5, Tet1/3^cDKO^ = 4 biological replicates) Data are mean ± SEM, unpaired *t* test. (**F**) Representative FACS plots and quantification of frequency and number of TEM/TCM, among GP33+ cells. (WT = 5, Tet1/3^cDKO^ = 4 biological replicates) Data are mean ± SEM, multiple unpaired *t* tests. (**G**) ELISA quantification of 5hmC in DNA isolated from the indicated cell types. (WT = 3, Tet1/3^cDKO^ biological replicates/ group). Data are mean ± SEM, One-Way ANOVA test. Data information: *P* values are indicated in the figures and *P* < 0.05 was considered significant; paired male or female mice were used and no gender biases associated with genotypes were observed. (**A**–**D**) Experiments were replicated at least two times. Source data are available online for this figure.

**Figure EV6:**
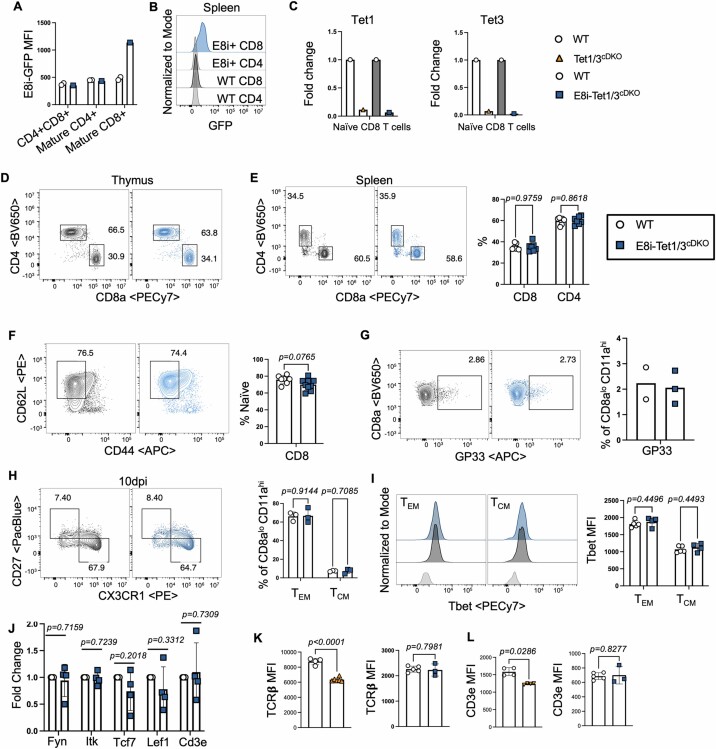
Tet1/3 are dispensable in peripheral CD8 T cells for effector and memory differentiation. (**A**) GFP MFI expressed in control Tet1/3fl/fl and E8iCreTgTet1/3fl/fl thymic populations. (WT = 2, Tet1/3^cDKO^ = 2 biological replicates). (**B**) Representative histograms showing GFP expression in control Tet1/3fl/fl- and E8iCreTgTet1/3fl/fl lymphocytes from the spleen (**C**) Fold change of Tet1 and Tet3 genomic copies in naive CD8 T cells from control Tet1/3fl/fl, Rorc(t)CreTgTet1/3fl/fl and E8iCreTgTet1/3fl/fl mice. (**D**) Representative FACS plots of CD4 and CD8 thymic T-cell populations from control Tet1/3fl/fl and E8iCreTgTet1/3fl/fl mice. Cells were pregated on LiveCD24-TCRb+CD69- cells. (**E**) Representative FACS plots and frequency of peripheral CD4 and CD8 splenic T-cell populations from control Tet1/3fl/fl and E8iCreTgTet1/3fl/fl mice. Cells were pregated on LiveTCRb+ cells. (WT = 7, Tet1/3^cDKO^ = 7 biological replicates). Data are mean ± SEM, multiple unpaired *t* tests. (**F**) Representative FACS plots and frequency of naive CD8 T cells from the lymph nodes and spleens of control Tet1/3fl/fl and E8iCreTgTet1/3fl/fl mice. Cells were pregated on LiveTCRb+CD8a+ cells. (WT = 7, Tet1/3^cDKO^ = 9 biological replicates). Data are mean ± SEM, unpaired t test. (**G**) Representative histograms and quantification of GP33-tetramer+ CD8 T cells 8 dpi. (WT = 2, Tet1/3^cDKO^ = 3 biological replicates). (**H**) Representation flow plots and quantification of TEM and TCM populations in the spleen 10 dpi. (WT = 3, Tet1/3^cDKO^ = 3 biological replicates) Data are mean ± SEM, multiple unpaired *t* tests. (**I**) Representative histograms and quantification of Tbet expression in TEM/TCM populations 30 dpi. (WT = 5, Tet1/3^cDKO^ = 4 biological replicates) Data are mean ± SEM, multiple unpaired tests. (**J**) Fold change of Fyn, Itk, Tcf, Lef1, Cd3e mRNA expression in activated CD8 T cells from control and E8iCreTgTet1/3fl/fl mice. Data is a summary of 2 independent experiments (WT = 4, Tet1/3cDKO = 4 biological replicates) Data are mean ± SEM, multiple unpaired *t* tests. (**K**) TCRb MFI on bulk CD8 + T cells from the spleen 8 dpi with LCMV-Armstrong. Data are mean ± SEM, unpaired *t* test. (WT = 4, RorcCreTet1/3^cDKO^ = 6 biological replicates; WT = 5, E8iCre-Tet1/3 ^cDKO^ = 3 biological replicates) Data are mean ± SEM, unpaired *t* test. (**L**) Cd3e MFI on bulk CD8 + T cells from the spleen 10 dpi with LCMV-Armstrong. Data are mean ± SEM, unpaired *t* test. (WT = 4, RorcCreTet1/3^cDKO^ = 4 biological replicates; WT = 5, E8iCre-Tet1/3 ^cDKO^ = 3 biological replicates) Data are mean ± SEM, unpaired *t* test. Data information: *P* values are indicated in the figures and *P* < 0.05 was considered significant; paired male or female mice were used and no gender biases associated with genotypes were observed. (**A**, **B**, **D**–**L**) Experiments were replicated at least two times. Source data are available online for this figure.

To determine whether the influence of Tet1/3 on CD8 effector fates stems from thymic programming of gene targets regulating effector T-cell differentiation or from their requirement during T-cell activation, we crossed Tet1/3 floxed mice onto an E8iCre-driver, in which Cre activity is observed in peripheral CD8^+^ T cells and activated CD8 T cells (Fig. EV6A,B), (Maekawa et al, 2008). We hypothesized that the deletion of Tet1/3 in mature CD8 T cells will not impact effector T-cell fates if thymic programming of the TCR-associated gene landscape is allowed to occur properly during lineage specification of CD8 T cells. We first confirmed genomic excision of Tet1/3 alleles by qPCR and found recombination efficiency of both alleles in sort-purified CD8 T cells from E8iCreTet1/3 floxed mice was as efficient as in sort-purified CD8 T cells from RorcCreTet1/3 floxed mice (Fig. EV6C). As expected, the thymic and peripheral CD4 and CD8 T-cell populations were unchanged in E8iCreTet1/3 floxed mice compared to controls (Fig. EV6D–F). In contrast to observations made in Rorc(t)Cre Tet1/3-deficient CD8 T cells, upon anti-CD3/CD28 mediated TCR stimulation, we observed no difference in IL-2 production in E8iCreTet1/3^fl/fl^ CD8 T cells compared to controls (Fig. 6A). In all, 8 dpi with LCMV-Armstrong, the proportion and total number of activated CD8 T cells in E8iCreTet1/3 floxed mice were comparable to WT controls (Fig. 6B). Moreover, the proportion of GP33-specific T cells were comparable to WT controls (Fig. EV6G) and no skewed differentiation into SLECs was observed (Fig. 6C) and upon ex vivo stimulation with GP33-41 peptide, E8iCreTet1/3^fl/fl^ CD8 T cells produced equal amounts of IFNγ as controls (Fig. 6D). Likewise, GP33-specific T_EM_ cell frequencies and numbers in E8iCreTet1/3 floxed mice was comparable to WT control mice 30 dpi (Figs. 6E,F and EV6H), in sharp contrast to observations made in Rorc(t)Cre Tet1/3 floxed mice (Fig. 1E). We confirmed T_EM/_T_CM_ memory fates via Tbet expression, in addition to surrogate CD27/CX3CR1 markers (Fig. EV6I). Furthermore, the gene expression of genes such as *Fyn, Itk* and *Cd3e* were not impaired in E8iCreTet1/3^fl/fl^ CD8 T cells (Fig. EV6J), nor was protein expression of TCRβ and Cd3e altered (Fig. EV6K,L) Lastly, to compare thymic programming via 5mC demethylation in developing CD8SP cells, we measured global 5hmC levels in CD8SP thymocytes FACS-purified from RorcCreTet1/3 floxed and E8iCreTet1/3 floxed mice. Consistent with our previous work and work from others (Issuree et al, 2018; Tsagaratou et al, 2014), we found an increase in 5hmC levels in CD8SP cells compared to DP thymocytes from WT mice. However, CD8SP from E8iCreTet1/3 floxed mice had comparable 5hmC to WT cells, while CD8SP cells from Rorc(t)Cre Tet1/3 floxed mice had significantly reduced 5hmC levels (Fig. 6G), in agreement with a critical role of Tet1/3 during lineage differentiation from DP precursors. Taken together, these data suggest that Tet1/3 are dispensable for effector and memory cell fates during peripheral CD8 T-cell priming but are key determinants of these fates via developmental programming.

## Discussion

T cells undergo dynamic changes in DNA methylation in key genes during effector and memory T-cell differentiation following an acute viral infection (Scharer et al, 2013; Youngblood et al, 2017). Understanding the importance of maintaining DNA methylation versus removing DNA methylation in the determination of T-cell lineage formation and function is key to developing context-appropriate therapeutic strategies in the clinic. Currently, our understanding of the role of DNA demethylation on T-cell fate outcomes during an acute infection remains incomplete. The ablation of Tet2 in developing T cells favors MPEC formation and the generation of T_CM_ cells following acute LCMV infection. Paradoxically however, ablation of Dnmt3a in activated CD8 T cells was also found to enhance the generation of MPECs and T_CM_ cells during acute LCMV infection (Youngblood et al, 2017). Furthermore, T cells also express Tet1 and Tet3. However, their roles in determining CD8 T-cell fates during acute viral infection remain unknown, adding complexity to our understanding of how the dynamics of methylation and demethylation impact T-cell fates.

In this study, we investigated the roles of Tet1/3 on CD8 effector and memory cell fate outcomes during acute LCMV infection. Surprisingly, we found that deletion of Tet1/3 in developing thymocytes resulted in increased generation of SLECs and T_EM_ cells following acute LCMV infection, in contrast to outcomes seen upon the deletion of Tet2 (Carty et al, 2018), underscoring the distinct and non-redundant roles of Tet1/3 in T cells. Remarkably, however, Tet1/3 were dispensable in peripheral CD8 T cells for SLEC/MPEC and T_EM_/T_CM_ cell fates following TCR activation. Instead, Tet1/3 were critically required for the epigenetic programming of a significant number of genes associated with regulation of the TCR-response pathway during thymic development. A failure to undergo DNA demethylation during thymic development did not alter the gross phenotypic characteristics of naive peripheral CD8 T cells. However, TCR stimulation unmasked selective alterations in effector programs in these cells, such as reduced IL-2 production and reduced TCR signaling strength. At a genomic level, T cells deficient in Tet1/3 displayed a significant decrease in chromatin accessibility within genes linked to the regulation of the TCR response as well as memory T-cell differentiation. Crucially, these differentially accessible regions were significantly enriched in motifs belonging to the bZIP TF family. AP-1 factors comprised of cFos and JunB heterodimers have previously been linked to binding almost 70% of early chromatin-accessible regions downstream of TCR signaling in human T cells (Yukawa et al, 2020). However, JunB binding was largely intact in Tet1/3-deficient CD8 T cells following TCR activation, implying that while DNA methylation hinders chromatin accessibility, it does not interfere with JunB binding. These results also suggest that JunB binding is not sufficient for promoting chromatin accessibility downstream of TCR stimulation and that increased binding of Bach factors, which compete for AP-1 binding sites to repress transcription (Jang et al, 2017; Roychoudhuri et al, 2016), are likely not the mechanisms behind reduced chromatin accessibility in activated Tet1/3-deficient T cells. Studies by others delving into how regulatory elements are poised for activation have proposed a model in which AP-1 transcription factors, in collaboration with lineage-specific transcription factors, engage with enhancers initially occluded by nucleosomes. This interaction recruits the SWI/SNF (BAF) remodeling complex, leading to nucleosome remodeling and the establishment of an accessible chromatin state (Vierbuchen et al, 2017; Yukawa et al, 2020). We thus propose a model whereby DNA methylation impedes either the recruitment of the BAF complex by AP-1 factors or interferes with nucleosome remodeling to allow for the activation of TCR-responsive elements that promote gene expression in effector T cells. Further studies are warranted to test this model.

Among key genes that regulate the TCR-response pathway, *Itk*, *Fyn*, and *Cd3e* were critical targets of DNA demethylation during thymic development, showing that the TCR-associated gene landscape is actively regulated by Tet1/3-dependent DNA demethylation. However, a limitation in our present study is that it is unclear whether the propensity of Tet1/3^cDKO^ T cells to differentiate into SLECs and T_EM_ cells is solely a consequence of altered regulation of the TCR-response pathway genes. Strong and sustained IL-2 signals promote effector and repress memory T-cell development (Kalia et al, 2010; Pipkin et al, 2010). However, autocrine IL-2 is dispensable for SLEC and T_EM_ differentiation (Toumi et al, 2022). Furthermore, considering that the co-transfer of WT P14 cells with Tet1/3cDKO cells into WT hosts failed to “rescue” the skewed SLEC and T_EM_ differentiation and that Tet3 cKO T cells did not show similar skewing despite the defect in IL-2, it is unlikely that autocrine IL-2 is a major driver of these phenotypes. Whether Tet1 plays a role in CD8 T-cell differentiation and the individual epigenetic contributions of Tet1 and Tet3 in this process are important areas of future investigations. Notably, although significant differences in methylation were seen in Tet1/3cDKO T cells, the catalytic-independent functions that these enzymes can have in this process cannot be ruled out (Ketchum et al, 2024; Xue et al, 2016).

Reduced TCR strength in T cells has previously been shown to increase the proportion of Ag-specific CD8 MPECs (Huang et al, 2015; Smith-Garvin et al, 2010). However, in the absence of Tet1/3, it is difficult to predict how net changes in TCR inputs regulated by a combination of various regulators that have both positive and negative feedback functions on TCR signaling affect effector and memory cell outcomes. Furthermore, increased effector memory cell fate outcomes in the absence of Tet1/3 may also be due to differences in transcription factors such as Tcf1 and Runx3 which are known regulators of central memory cell fate (Jeannet et al, 2010; Pais Ferreira et al, 2020; Wang et al, 2018; Zhou et al, 2010). Nevertheless, our findings depict a novel and important epigenetic mechanism by which the TCR-regulatory landscape in peripheral naive T cells is fine-tuned during development and raise exciting questions about the importance of this mechanism in T-cell immunity and tolerance during aging and stress, where dramatic changes in metabolism can lead to epigenetic alterations in developing T cells and have an impact on vaccination outcomes (Moskowitz et al, 2017; Palmer, 2013; Soto-Heredero et al, 2023). Our results using tetramers to examine clonal populations of CD8 T cells during infection hint that Tet1/3 deficiency may alter the TCR repertoire or immunodominance during an acute immune response, although further investigations are necessary. They also suggest that memory T_EM_ and T_CM_ populations generated in the absence of Tet1/3 do not persist for long, as they were significantly reduced 24 dpi. Future studies examining the recall potential of these cells following a secondary immune challenge will shed light on the importance of Tet1/3 in memory T cells.

The combined activities of Tet2 and Tet3 were previously found to be critical for iNKT T-cell differentiation (Tsagaratou et al, 2017), suggesting that Tet2 is required during thymic T-cell development. Consequently, the possibility that Tet2 deletion in developing T cells leads to increased MPECs and T_CM_ cells because of aberrant epigenetic programming in the thymus cannot be discounted. Revisiting the role of Tet2 in peripheral CD8 T-cell differentiation will be important to reconcile the paradoxical observations that loss of either Dnmt3a or Tet2 results in similar cell fate outcomes during acute LCMV infection. Alternatively, it is plausible that Dnmt3a and Tet2 cooperate to regulate distinct transcriptional nodes necessary for MPEC and T_CM_ T-cell differentiation, as a precedent for such cooperative programs exist in other systems (Zhang et al, 2016). Lastly, although we found that Tet1/3 were dispensable for effector and memory cell differentiation, it remains to be seen whether they play a role in regulating recall memory responses. Future investigations examining changes in DNA methylation in memory CD8 T cells lacking Tet1/3 using the E8iCre mouse model system will provide valuable insights into the gene networks influenced by DNA demethylation in this process. These inquiries will be crucial for anticipating the impact of demethylation on shaping the immune gene landscape during vaccination and will open novel avenues that can be harnessed to strategically influence vaccination outcomes.

## Methods

**Table:** Reagents and tools table

Reagent/resource	Reference or source	Identifier or catalog number
**Experimental models**		
RorcCreTg1Litt/J	Jackson Labs	22791
Tet1 floxed (C57BL/6J)	Gift from Dr. Aifantis (NYU)	NA
Tet3 floxed mice (129 background)	Gift from Dr. Yi Zhang (Harvard)	NA
P14Tg (Thy1.2/Thy1.1)	Gift from Dr. Noah Butler (University of Iowa)	NA
Rorc(t)Cre Tet1/3 floxed	This paper; Tet1/3 were backcrossed onto C57BL/6 J > 6 generations	NA
E8iCreTg	Gift from Dr. Josalyn Cho; available at Jackson Labs	8766
B6.SJL-CD45.1	Jackson Labs	002014
**Antibodies**		
APC-Cy7 anti-mouse CD45.1 (clone A20)	Tonbo	Cat#: 70-0453, RRID:AB_2621497
BV650 anti-mouse CD4 (clone RM4-5)	Biolegend	Cat#: 100555, RRID:AB_2562098
FITC anti-mouse CD8a (clone 53-6.7)	Biolegend	Cat#: 100706, RRID:AB_312745
BV650 anti-mouse CD8a (clone 53.6.7)	Biolegend	Cat#: 100742, RRID:AB_2563056
PerCp5.5 anti-mouse CD8a (clone QA17A07)	Biolegend	Cat#: 155014, RRID:AB_2890703
PECy7 anti-mouse CD8a (clone 53.6.7)	Biolegend	Cat#: 100722, RRID:AB_312760
PerCp5.5 anti-mouse TCRb (clone H57-597)	Tonbo	Cat#: 65-5961, RRID:AB_2621911
vF450 anti-mouse CD24 (clone M1/69)	Tonbo	Cat#: 75-0242
PE anti-mouse CD25 (clone PC61.5)	Biolegend	Cat#: 102007, RRID:AB_312857
PerCp5.5 anti-mouse CD19 (clone 1D.3)	Tonbo	Cat#: 65-0193, RRID:AB_2621887
FITC anti-mouse CD62L (clone MEL-14)	Tonbo	Cat#: 35-0621, RRID:AB_2621697
PE anti-mouse CD62L (clone W18081D)	Biolegend	Cat#: 161204, RRID:AB_2876576
vF500 anti-mouse CD44 (clone IM7)	Tonbo	Cat#: 85-0441
APC anti-mouse CD44 (clone IM7)	Biolegend	Cat#: 103012, RRID:AB_312962
PE anti-mouse CD69 (clone H1.2F3)	Tonbo	Cat#: 50-0691
APC anti-mouse CD11a (clone M17/4)	Biolegend	Cat#: 101120, RRID:AB_2562779
FITC anti-mouse CD11a (clone M17/4)	Biolegend	Cat#: 101106, RRID:AB_312779
PE anti-mouse CD11a (clone M17/4)	Tonbo	Cat#: 50-0111
BV421 anti-mouse KLRG1 (clone 2F1-KLRG1)	Biolegend	Cat#: 75-5893, RRID:AB_2621966
PECy7 anti-mouse CD127 (clone A7R34)	Tonbo	Cat#: 60-1271, RRID:AB_2621859
FITC CD90.1 anti-mouse (clone OX-7)	Biolegend	Cat#: 202503, RRID:AB_314014
PE CD90.2 anti-mouse (clone 30-H12)	Biolegend	Cat#: 105308, RRID:AB_313179
PE/Dazzle 594 anti-mouse CD90.2 (clone 30-H12)	Biolegend	Cat#: 105340, RRID:AB_2632886
eF450 CD90.2 anti-mouse (clone 30-H12)	Invitrogen	Cat#: 48-0902-82, RRID:AB_1272200
PacBlue anti-mouse CD27 (clone LG.3A10)	Biolegend	Cat#: 124218, RRID:AB_2561546
FITC anti-mouse CX3CR1 (clone SA011F11)	Biolegend	Cat#: 149020, RRID:AB_2565703
PE anti-mouse CX3CR1 (clone SA011F11)	Biolegend	Cat#: 153706, RRID:AB_2734221
PECy7 anti-mouse IL-2 (clone JES6-5H3)	Biolegend	Cat#: 503832, RRID:AB_2561750
APC anti-mouse IFN-g (clone XMG1.2)	Tonbo	Cat#: 20-7311, RRID:AB_2621616
BV421 anti-mouse TNFa (cloneMP6-XT22)	Biolegend	Cat#: 506327, RRID:AB_10900823
FITC anti-mouse Nur77 (clone 12.14)	Invitrogen	Cat#: 53-5965-82, RRID:AB_2574429
PE anti-mouse Nur77 (clone 12.14)	Invitrogen	Cat#: 12-5965-82, RRID:AB_1257209
PECy7 anti-mouse Tbet (clone 4B10)	Biolegend	Cat#: 644824, RRID:AB_2561761
PE/Dazzle 594 Ki67 (clone 11F6)	Biolegend	Cat#: 151219, RRID:AB_2910306
AffiniPure Goat Anti-Armenian Hamster IgG	Jackson ImmunoResearch Labs	Cat#: 127-005-099, RRID:AB_2338971
Anti-Mouse CD28 Purified	Tonbo	Cat#: 70-0281-U025
Anti-Mouse CD3 Purified	Tonbo	Cat#: 70-0031-U100
pERK	Cell Signaling	Cat#: 4370 T
B-actin	DSHB	Cat#: 224-236-1
JunB	Cell Signaling	Cat#: 3753 T
**Oligonucleotides and other sequence-based reagents**		
Hprt Forward: 5’-GTT GGG CTT ACC TCA CTG CT-3’	This study	
Hprt Reverse: 5’-TCA TCG CTA ATC ACG ACG CT-3’	This study	
Cd8a promoter Forward: 5’-TCT GCA AGG GTG CAT TCT CAC TCT-3’	This study	
Cd8a promoter Reverse: 5’-AGC TGC AGA CAG AGC TGA TTT CCT-3’	This study	
Il2 Forward: 5’-TGA ACT TGG ACC TCT GCG G-3’	This study	
Il2 Reverse: 5’-TGT GTT GTC AGA GCC CTT TAG T-3’	This study	
Tet1 Forward: 5’-GTC AGG GAG CTC ATG GAG AC-3’	This study	
Tet1 Reverse: 5’-CCT GAG AGC TCT TCC CTT CC-3’	This study	
Tet2 Forward: 5’-AAC CTG GCT ACT GTC ATT GCT CCA-3’	This study	
Tet2 Reverse: 5’-ATC TTC TGC TGG TCT CTG TGG GAA-3’	This study	
Tet3 Forward: 5’-AAC GGC TGC AAA TAT GCT CG-3’	This study	
Tet3 Reverse: 5’-TCC TCC TCC TTC GGA TTG TCT-3’	This study	
Junb Forward: 5’-AGG CAG CTA CTT TTC GGG TC-3’	This study	
Junb Reverse: 5’-TTG CTG TTG GGG ACG ATC AA-3’	This study	
Itk Forward: 5’-GGT GTC CGA CTT TGG GAT GA-3’	This study	
Itk Reverse: 5’-AGG AGA ACA CCT CTG GGG AT-3’	This study	
Cd3e Forward: 5’-TGC TAC ACA CCA GCC TCA AAT-3’	This study	
Cd3e Reverse: 5’-CAG CAA GCC CAG AGT GAT ACA-3’	This study	
Fyn Forward: 5’-AAG CAC GGA CGG AAG ATG AC-3’	This study	
Fyn Reverse: 5’-ATG GAG TCA ACT GGA GCC AC-3’	This study	
Tcf7 Forward: 5’-CTG TCC CCT TCC TGC GGA T-3’	This study	
Tcf7 Reverse: 5’-TGT CCA GGT ACA CCA GAT CCC-3’	This study	
Lef1 Forward: 5’-ACT GTC AGG CGA CAC TTC CAT G-3’	This study	
Lef1 Reverse: 5’-GTG CTC CTG TTT GAC CTG AGG T-3’	This study	
**Chemicals, enzymes, and other reagents**		
SsoAdvanced Universal SYBR Green Supermix	Bio-Rad	Cat#: 1725271
LCMV gp33-41 peptide	GenScript	Cat#: RP20257
CFSE	Invitrogen	Cat#:C34554
Ghost Dye Violet 510 Viability Dye	Tonbo	Cat#: 13-0870-T100
Ghost Dye Red 710 Viability Dye	Tonbo	Cat#: 13-0871-T100
Zombie NIR Dye	Biolegend	Cat#: 423105
BD OptEIA Mouse IFN-g ELISA Set	BD Biosciences	Cat#: 555138
BD OptEIA Mouse IL-2 ELISA Set	BD Biosciences	Cat#: 555148
Dynabeads Untouched CD8 Cells	Invitrogen	Ref#: 11417D
Foxp3/Transcription Factor Staining Buffer Kit	Cytek	Ref#: TNB-0607-KIT
RNeasy MiniElute Cleanup Kit	Qiagen	Ref#: 74204
iScript cDNA Synthesis Kit	Bio-Rad	Cat#: 1708890
KAPA Express Extract	Roche	Cat#: 50-196-5275
DNeasy Blood & Tissue Kit	Qiagen	Cat#: 69504
NEBNext Enzymatic Methyl-seq Kit	New England Biolabs	Cat#: E7120S
ATAC-Seq Kit	Active Motif	Cat#: 53150
CUT&Tag-IT Assay Kit – Cells	Active Motif	Cat#: 53160
**Software**		
Prism8	Graphpad	NA
BioRender	NA	NA
Bowtie 2 version 2.4.1	10.1038/nmeth.1923	N/A
MACS2 v2.2.7.1	10.18129/B9.bioc.MACSr	N/A
ChIPseeker v1.28.3	10.18129/B9.bioc.ChIPseeker	N/A
ChIPpeakAnno v3.32	10.18129/B9.bioc.ChIPpeakAnno	N/A
Trimmomatic-v0.35	10.1093/bioinformatics/btu170	N/A
HOMER	http://homer.ucsd.edu/homer/motif/	N/A
methylKit version 1.16.1	10.18129/B9.bioc.methylKit	N/A
DiffBind version 2.10.0	10.18129/B9.bioc.DiffBind	N/A
Bismark v0.23.1	10.1093/bioinformatics/btr167	N/A
SeqMonk v1.48.1	https://www.bioinformatics.babraham.ac.uk/projects/download.html#seqmonk	N/A
Flow jo (v10.8.2)	BeckmanCoulter	N/A
**Other**		
Illumina NovaSeq 6000	Illumina	

### Mice

Tet1 floxed (C57BL/6J) and Tet3 floxed mice (129 backcrossed to C57BL/6J) were kindly provided by Dr. Iannis Aifantis (Moran-Crusio et al, 2011) and Dr. Yi Zhang (Shen et al, 2014), respectively. P14Tg (Thy1.2/Thy1.1) transgenic mice on a C57BL/6 background were generously provided by Dr. Noah Butler. These mice were then backcrossed onto RorcCreTg mice (Jax Lab B6.FVB-Tg(RorcCreTg)1Litt/J #022791). Thy1.1/Thy1.1 homozygous P14-RorcCreTg Tet1/3 floxed mice were generated and maintained in-house. E8iCreTg mice (Jax Lab #008766) were kindly provided by Dr. Josalyn Cho to generate E8iCreTgTet1 and Tet3 floxed mice. B6.SJL-CD45.1 mice were purchased from the Jackson Laboratory (#002014). All mice were maintained under specific pathogen-free (SPF) conditions at the barrier animal facility at the University of Iowa Carver College of Medicine on a 12-h light cycle at 30–70% humidity and temperature of 20–26 °C, with access to standard chow and water. Littermate controls (No Cre Tet1/3 floxed), and sex-matched 6-8-week-old mice were used for all experiments unless specified. CO_2_ exposure was used as the method for euthanasia for all experiments. Permission for all animal experiments performed was granted by the IACUC at the University of Iowa Carver College of Medicine.

### Naive T-cell sorting

Single-cell suspensions were prepared from the spleen and/or lymph nodes after mashing through 70 µm cell strainers. Red blood cells were lysed in ACK lysis Buffer (Gibco), and a Dynabeads Untouched Mouse T-cell kit (Thermo Fisher) was used to enrich for CD4^+^ and CD8^+^ T cells. Cells were surface-stained in IMDM containing 2% FBS for 30 min at 4 °C. Cells were washed, and naive CD8 + T cells were then FACS Sorted on a BD FACSAria II or FACSAria Fusion cell sorter by gating on CD19^-^CD25^-^CD8^+^CD62L^hi^CD44^−^ T cells. Cell purity was verified and estimated to be >98%.

### Flow cytometry analysis

For immune subset profiling, single-cell suspensions were prepared from the thymus, spleen, peripheral lymph nodes or mesenteric lymph nodes by mashing through 70-µm cell strainers. Red blood cells were lysed in ACK lysis Buffer (Gibco) and counted on a Countess II Automated Cell Counter (Thermo Fisher). Cells were surface-stained in IMDM containing 2% FBS for 30 min at 4 °C or 20 min at RT followed by fixation with a Foxp3/TF Staining Buffer Kit (Tonbo). Cells were then permeabilized and stained for TFs (1 h, RT). For intracellular detection of cytokine production, cells were incubated in the presence of Monensin and Brefeldin A for 5–6 h at 37 °C, followed by staining. Stained cells were analyzed on a Cytoflex flow analyzer or an LSR-II flow analyzer. Flow jo (v10.8.2) was used for all flow cytometry analysis.

### Antibodies

The antibodies used for flow cytometry and cell sorting were purchased from BioLegend, BD Biosciences, Thermo Fisher Scientific, or Tonbo Biosciences and their clone numbers are CD45.1 (A20), CD4 (RM4-5), CD8α (53-6.7), TCRβ (H57-597), CD24 (M1/69), CD25 (PC61.5), CD19 (1D.3) CD62L (MEL-14), CD44 (IM7), CD69 (H1.2F3), CD11a (M17/4), KLRG1 (2F1-KLRG1), CD127 (A7R34), CD90.1 (OX-7), CD90.2 (30-H12), CD27 (LG.3A10), CX3CR1 (SA011F11), IL-2 (JES6-5H3), Nur77 (12.14), IFNγ (XMG1.2), Tbet (4B10), Ki67 (16A8). Ghost dye was used for the exclusion of dead cells. For further details, please see the Reagents and Tools Table.

### Quantitative real-time reverse transcription

DNase I-treated total RNA was prepared from both naive and activated T cells using RNeasy RNA isolation kit (Qiagen) and cDNA was synthesized using an iScript cDNA synthesis kit (Biorad). Tet1 and Tet3 excision efficiency was assessed following lysis using KAPA Express Extract (Roche). Quantitative PCR was performed using SsoAdvanced Universal SYBR Green supermix (Biorad) and a CFX Connect Real-time PCR detection system (Biorad). The following primers were used for qRT-PCR.

### In vitro activation of naive CD8 + T cells (anti-CD3/CD28)

Flat-bottom tissue culture plates were coated with polyclonal goat affinity-purified antibody to hamster IgG (MP Biomedical). FACS-sorted CD19-CD25-CD8+CD62LhiCD44- naive T cells were seeded in T-cell medium [RPMI 1640 (Gibco), 10% heat-inactivated FBS (RγD), 2 mM l-glutamine, 50 µg/ml gentamicin, 1% Penn/Strep, and 50 µM 2-mercaptoethanol (Gibco)] on IgG-bound plates along with 0.1 µg/mL anti-CD3 (Tonbo, clone 17A2) and 0.025 µg/mL anti-CD28 (Tonbo, clone 37.51) antibodies. At indicated timepoints, supernatants were collected for subsequent analysis and cells were mechanically lifted off the plates, allowed to rest for 30 min at 37 °C and surface markers were stained for live assessment by flow cytometry or fixed as described above for subsequent staining. CFSE labeling for certain experiments was performed prior to seeding on tissue culture plates.

### In vitro activation of P14 T cells

Collagenase D-treated LNs and spleens were mashed through 70-µm cell strainers, red blood cells (RBCs) were lysed in ACK lysis Buffer (Gibco). Antigen presenting cells and P14-RorcCreTg CD8 T cells were seeded in T-cell medium with LCMV gp33-41 peptide (GenScript) and 0.25 µg/mL anti-CD28 (Tonbo, clone 37.51) antibody.

### Adoptive transfer of P14 T cells and LCMV infection

Spleens from P14 mice were mashed through 70 µm cell strainer, RBCs were lysed. An aliquot of cells was surface-stained to characterize the frequency of TCRβ^+^CD8^+^ T cells by flow cytometry. Cell numbers were adjusted such that 5000 WT and 5000 Tet1/3^cDKO^ P14 T cells in 200 µL volume were transferred by i.v. injection into CD45.1 recipients. Mice were challenged with a standard dose of LCMV-Armstrong (2.0 × 10^5^ PFU) by i.p. injection one day following adoptive T-cell transfer.

### ELISA

IL-2 and IFNγ measurements in the supernatants were performed using a BD OptEIA mouse IL-2 ELISA Set and BD OptEIA mouse IFNγ ELISA Set (BD Biosciences) following the manufacturer’s instructions. Supernatants from anti-CD3/anti-CD28 and gp33-41 peptide-stimulated T cells were diluted between 10 and 100-fold in diluent prior to the ELISA, and undiluted supernatants from unstimulated T cells were used as controls. ELISAs were read at OD450 on SpectraMax 384 Plus spectrophotometer. Cytokine levels were calculated through extrapolation from a standard curve using Excel. 5hmC ELISA was performed using the Global 5hmC DNA ELISA kit (Active Motif), and 25 ng of DNA/sample was used. Technical duplicates were run for each sample.

### Whole-genome enzymatic methyl sequencing (EM-Seq) sample processing

Genomic DNA was isolated from naive sorted cells or from sorted CD8 T cells stimulated for 18 h in vitro using a DNA Extraction Kit (Qiagen) and sonicated to generate fragments of ~350 to 400 base pairs using the Bioruptor Pico sonication device (Diagenode). Unmethylated cytosines were converted to uracils, and sequencing libraries were created using the NEBnext Enzymatic Methyl Seq Kit (New England Biolabs) according to the manufacturer’s instructions. DNA libraries were sequenced using an Illumina NovaSeq 6000 system following the manufacturer’s protocols.

### Genome-wide methylation data analysis

Sequencing data quality was assessed using FastQC v0.11.4. Adapters were trimmed from the sequencing reads using Trimmomatic-v0.39 (Bolger et al, 2014) using options (java -jar trimmomatic-0.39.jar PE -phred33 $FORWARD_READS$REVERSE_READSIILLUMINACLIP:NexteraPE:2:30:10:2:keepBothReads LEADING:3 TRAILING:3 AVGQUAL:20 MINLEN:35). Alignment to the GRCm39/mm39 reference genome was performed using Bismark v0.23.1 (Krueger and Andrews, 2011) with options (bismark GRCm39 -1 $FORWARD_READS -2 $REVERSE_READS). Deduplication was performed with deduplicate_bismark (deduplicate_bismark $BISMARK_ALIGNED_BAM). Library quality was assessed based on the percentage of reads that aligned to the genome. Library quality was considered sufficient if greater than 50% of reads uniquely aligned to the genome. Enzymatic methyl conversion efficiency was assessed by evaluating the percent of methylation observed in the CHH genome context. Enzymatic methyl conversion was considered sufficient when this value was less than 3%. Genome coverage was assessed using the bedtools genomecov software v2.25.0. Library genome coverage was considered sufficient if 80% of the genome had a depth of at least 10 reads. For each library that met these quality metrics, methylation percentages at individual CpG positions in the reference genome were quantified using the Bismark Methylation Extractor program (Krueger and Andrews, 2011) with options (Bismark-0.23.1/bismark_methylation_extractor --bedGraph --paired-end $BISMARK_DEDUPLICATED_BAM). Differentially methylated loci among the datasets were detected using SeqMonk v1.48.1, and each “Probe” was defined to be the gene body +3 kb upstream (“promoter region”) (www.bioinformatics.babraham.ac.uk). Differential methylation analysis was then performed via logistic regression using the R package methylKit version 1.16.1 (Akalin et al, 2012). Visualization of CpG positions on colored heatmaps (white-red) reflects the percent methylated from 0 to 100%. Individual genomic loci were displayed using UCSC Genome Browser (52). Enrichment analysis to identify pathways and transcription factors associated with hypermethylated genes was performed using Enrichr (Xie et al, 2021).

### ATAC-Seq sample processing

ATAC-Seq was performed using ~90,000 cells/sample using an ATAC‐Seq Kit (Active Motif) following the manufacturer’s protocol. Naive CD8 T cells were sorted and stimulated as described above for 18 h. T cells were mechanically lifted and allowed to rest for 20 min prior to processing for ATAC-Seq. P14 CD8 T cells were harvested 5 dpi for ATAC-Seq by sorting on CD45.1-CD8a + CD11a + CD90.1/CD90.2 cells. After purification of tagmented DNA, a quantitative qPCR determined that on average 8 cycles were required for library amplification. The library was purified, and size was selected with Agencourt AMPure beads to remove >2000-bp fragments and excess primers. Samples underwent quality control and quantification on an Agilent BioAnalyzer by the Genomics core at the University of Iowa before pooling and sequencing on an Illumina Novaseq 6000.

### Cut and Tag sample processing

Activated T cells were harvested following 18 h stimulation, counted, and centrifuged for 3 min at 600 × g at room temperature, and 300,000–500,000 cells per condition were used. Cells were processed for Cut and Tag using a Cut and Tag kit (Active Motif) using the manufacturer’s protocol. A commercially available Rabbit JunB antibody (Clone C37F9) from Cell Signaling was used. Libraries were purified, and size selection was performed with Agencourt AMPure beads to remove >2000-bp fragments and excess primers.

### ATAC-Seq and Cut and Tag data analysis

Sequencing adapters were removed from paired‐end reads using Trimmomatic version 0.39 (Bolger et al, 2014). Trimmed reads were aligned to mouse reference genome GRCm38/mm10 using Bowtie 2 version 2.4.1 with options ‐‐very‐sensitive ‐X 2000. Following genome alignment, Picard was used to mark PCR duplicates, and Samtools version 1.3.1 was used to remove PCR duplicates, discordant pairs, and alignments to the mitochondrial genome. Peak calling was performed using MACS2 version 2.2.7.1, and differential accessibility analyses of MACS2 peaks were performed using the R package DiffBind version 2.10.0. Differentially accessible peaks were annotated to genome regions of interest using the R package ChIPseeker version 1.18.0, and sequence motif enrichment analyses were performed using HOMER version 4.11.1. A cut-off of 200 bp from the peak detected was used for motif enrichment analysis. Gene ontology analysis was performed using Enrichr (Xie et al, 2021).

### Statistical analysis

Graph Pad Prism (v. 9.0 or 10) was used for all statistical analysis. For comparison between two experimental groups, unpaired *t* test or paired Wilcoxon signed-rank test was used. For multiple comparisons, two-way ANOVA Fishers LSD test were used with indicated multiple comparison on the figure to determine the statistical significance between two specific groups. *P* values of ≤0.05 are considered statistically significant and *P* values are indicated on the figures. All experiments were replicated independently two to three times.

## Supplementary information

Peer Review File

Dataset EV1

Dataset EV2

Dataset EV3

Dataset EV4

Dataset EV5

Figure EV Source Data

Source data Fig. 1

Source data Fig. 2

Source data Fig. 3

Source data Fig. 5

Source data Fig. 6

Expanded View Figures

## Data Availability

ATAC-seq, Cut and Run, and EM-Seq sequencing files from this work have been deposited to the Gene Expression Omnibus database with accession number GSE252583. Files associated with the main figures of the manuscript have been deposited on DataDryad at https://datadryad.org/share/LkVxTE1twDjO50Vab1hhZJsblYgfDW_uPpJmZEeA02I. The source data of this paper are collected in the following database record: biostudies:S-SCDT-10_1038-S44319-025-00439-z.

## References

[CR1] Akalin A, Kormaksson M, Li S, Garrett-Bakelman FE, Figueroa ME, Melnick A, Mason CE (2012) methylKit: a comprehensive R package for the analysis of genome-wide DNA methylation profiles. Genome Biol 13:R87 23034086 10.1186/gb-2012-13-10-r87 PMC3491415

[CR2] Altan-Bonnet G, Germain RN (2005) Modeling T cell antigen discrimination based on feedback control of digital ERK responses. PLoS Biol 3:e356 16231973 10.1371/journal.pbio.0030356 PMC1262625

[CR3] Andreotti AH, Schwartzberg PL, Joseph RE, Berg LJ (2010) T-cell signaling regulated by the Tec family kinase, Itk. Cold Spring Harb Perspect Biol 2:a002287 20519342 10.1101/cshperspect.a002287 PMC2890196

[CR4] Ashouri JF, Weiss A (2017) Endogenous Nur77 is a specific indicator of antigen receptor signaling in human T and B cells. J Immunol 198:657–668 27940659 10.4049/jimmunol.1601301 PMC5224971

[CR5] Bending D, Prieto Martin P, Paduraru A, Ducker C, Marzaganov E, Laviron M, Kitano S, Miyachi H, Crompton T, Ono M (2018) A timer for analyzing temporally dynamic changes in transcription during differentiation in vivo. J Cell Biol 217:2931–2950 29941474 10.1083/jcb.201711048 PMC6080944

[CR6] Bevington SL, Cauchy P, Piper J, Bertrand E, Lalli N, Jarvis RC, Gilding LN, Ott S, Bonifer C, Cockerill PN (2016) Inducible chromatin priming is associated with the establishment of immunological memory in T cells. EMBO J 35:515–535 26796577 10.15252/embj.201592534 PMC4772849

[CR7] Bolger AM, Lohse M, Usadel B (2014) Trimmomatic: a flexible trimmer for Illumina sequence data. Bioinformatics 30:2114–2120 24695404 10.1093/bioinformatics/btu170 PMC4103590

[CR8] Buenrostro JD, Wu B, Chang HY, Greenleaf WJ (2015) ATAC-seq: a method for assaying chromatin accessibility genome-wide. Curr Protoc Mol Biol 109:21 29 21–21 29 29 10.1002/0471142727.mb2129s109 PMC4374986 25559105

[CR9] Carty SA, Gohil M, Banks LB, Cotton RM, Johnson ME, Stelekati E, Wells AD, Wherry EJ, Koretzky GA, Jordan MS (2018) The loss of TET2 promotes CD8(+) T cell memory differentiation. J Immunol 200:82–91 29150566 10.4049/jimmunol.1700559 PMC5736442

[CR10] Cedar H, Sabag O, Reizel Y (2022) The role of DNA methylation in genome-wide gene regulation during development. Development 149:dev200118 35051273 10.1242/dev.200118

[CR11] Chatila T, Silverman L, Miller R, Geha R (1989) Mechanisms of T cell activation by the calcium ionophore ionomycin. J Immunol 143:1283–1289 2545785

[CR12] Chin SS, Guillen E, Chorro L, Achar S, Ng K, Oberle S, Alfei F, Zehn D, Altan-Bonnet G, Delahaye F, Lauvau G (2022) T cell receptor and IL-2 signaling strength control memory CD8(+) T cell functional fitness via chromatin remodeling. Nat Commun 13:2240 35474218 10.1038/s41467-022-29718-2 PMC9042912

[CR13] Chung HK, McDonald B, Kaech SM (2021) The architectural design of CD8+ T cell responses in acute and chronic infection: parallel structures with divergent fates. J Exp Med 218:e20201730 33755719 10.1084/jem.20201730 PMC7992501

[CR14] Daniels MA, Teixeiro E (2015) TCR signaling in T cell memory. Front Immunol 6:617 26697013 10.3389/fimmu.2015.00617 PMC4674549

[CR15] Eberl G, Littman DR (2004) Thymic origin of intestinal alphabeta T cells revealed by fate mapping of RORgammat+ cells. Science 305:248–251 15247480 10.1126/science.1096472

[CR16] Gallagher MP, Conley JM, Vangala P, Garber M, Reboldi A, Berg LJ (2021) Hierarchy of signaling thresholds downstream of the T cell receptor and the Tec kinase ITK. Proc Natl Acad Sci USA 118:e2025825118 34452995 10.1073/pnas.2025825118 PMC8536361

[CR17] Gerlach C, Moseman EA, Loughhead SM, Alvarez D, Zwijnenburg AJ, Waanders L, Garg R, de la Torre JC, von Andrian UH (2016) The chemokine receptor CX3CR1 defines three antigen-experienced CD8 T cell subsets with distinct roles in immune surveillance and homeostasis. Immunity 45:1270–1284 27939671 10.1016/j.immuni.2016.10.018 PMC5177508

[CR18] Gerlach C, Rohr JC, Perie L, van Rooij N, van Heijst JW, Velds A, Urbanus J, Naik SH, Jacobs H, Beltman JB et al (2013) Heterogeneous differentiation patterns of individual CD8+ T cells. Science 340:635–639 23493421 10.1126/science.1235487

[CR19] Glover JN, Harrison SC (1995) Crystal structure of the heterodimeric bZIP transcription factor c-Fos-c-Jun bound to DNA. Nature 373:257–261 7816143 10.1038/373257a0

[CR20] Greenberg MVC, Bourc’his D (2019) The diverse roles of DNA methylation in mammalian development and disease. Nat Rev Mol Cell Biol 20:590–607 31399642 10.1038/s41580-019-0159-6

[CR21] Harty JT, Tvinnereim AR, White DW (2000) CD8+ T cell effector mechanisms in resistance to infection. Annu Rev Immunol 18:275–308 10837060 10.1146/annurev.immunol.18.1.275

[CR22] Huang F, Huang W, Briggs J, Chew T, Bai Y, Deol S, August A (2015) The tyrosine kinase Itk suppresses CD8+ memory T cell development in response to bacterial infection. Sci Rep. 5:7688 25567129 10.1038/srep07688 PMC4286740

[CR23] Issuree PD, Day K, Au C, Raviram R, Zappile P, Skok JA, Xue HH, Myers RM, Littman DR (2018) Stage-specific epigenetic regulation of CD4 expression by coordinated enhancer elements during T cell development. Nat Commun 9:3594 30185805 10.1038/s41467-018-05834-w PMC6125341

[CR24] Jang E, Lee HR, Lee GH, Oh AR, Cha JY, Igarashi K, Youn J (2017) Bach2 represses the AP-1-driven induction of interleukin-2 gene transcription in CD4+ T cells. BMB Rep. 50:472–477 28855027 10.5483/BMBRep.2017.50.9.124 PMC5625695

[CR25] Janulis M, Silberman S, Ambegaokar A, Gutkind JS, Schultz RM (1999) Role of mitogen-activated protein kinases and c-Jun/AP-1 trans-activating activity in the regulation of protease mRNAs and the malignant phenotype in NIH 3T3 fibroblasts. J Biol Chem 274:801–813 9873019 10.1074/jbc.274.2.801

[CR26] Jeannet G, Boudousquie C, Gardiol N, Kang J, Huelsken J, Held W (2010) Essential role of the Wnt pathway effector Tcf-1 for the establishment of functional CD8 T cell memory. Proc Natl Acad Sci USA 107:9777–9782 20457902 10.1073/pnas.0914127107 PMC2906901

[CR27] Jones PA (2012) Functions of DNA methylation: islands, start sites, gene bodies and beyond. Nat Rev Genet 13:484–492 22641018 10.1038/nrg3230

[CR28] Joshi NS, Cui W, Chandele A, Lee HK, Urso DR, Hagman J, Gapin L, Kaech SM (2007) Inflammation directs memory precursor and short-lived effector CD8(+) T cell fates via the graded expression of T-bet transcription factor. Immunity 27:281–295 17723218 10.1016/j.immuni.2007.07.010 PMC2034442

[CR29] Kalia V, Sarkar S, Subramaniam S, Haining WN, Smith KA, Ahmed R (2010) Prolonged interleukin-2Ralpha expression on virus-specific CD8+ T cells favors terminal-effector differentiation in vivo. Immunity 32:91–103 20096608 10.1016/j.immuni.2009.11.010

[CR30] Katagiri T, Yamazaki S, Fukui Y, Aoki K, Yagita H, Nishina T, Mikami T, Katagiri S, Shiraishi A, Kimura S et al (2019) JunB plays a crucial role in development of regulatory T cells by promoting IL-2 signaling. Mucosal Immunol 12:1104–1117 31285535 10.1038/s41385-019-0182-0

[CR31] Kaya-Okur HS, Wu SJ, Codomo CA, Pledger ES, Bryson TD, Henikoff JG, Ahmad K, Henikoff S (2019) CUT&Tag for efficient epigenomic profiling of small samples and single cells. Nat Commun 10:1930 31036827 10.1038/s41467-019-09982-5 PMC6488672

[CR32] Ketchum HC, Suzuki M, Dawlaty MM (2024) Catalytic-dependent and -independent roles of TET3 in the regulation of specific genetic programs during neuroectoderm specification. Commun Biol 7:415 38580843 10.1038/s42003-024-06120-w PMC10997653

[CR33] Krueger F, Andrews SR (2011) Bismark: a flexible aligner and methylation caller for Bisulfite-Seq applications. Bioinformatics 27:1571–1572 21493656 10.1093/bioinformatics/btr167 PMC3102221

[CR34] Ladle BH, Li KP, Phillips MJ, Pucsek AB, Haile A, Powell JD, Jaffee EM, Hildeman DA, Gamper CJ (2016) De novo DNA methylation by DNA methyltransferase 3a controls early effector CD8+ T-cell fate decisions following activation. Proc Natl Acad Sci USA 113:10631–10636 27582468 10.1073/pnas.1524490113 PMC5035851

[CR35] Maekawa Y, Minato Y, Ishifune C, Kurihara T, Kitamura A, Kojima H, Yagita H, Sakata-Yanagimoto M, Saito T, Taniuchi I et al (2008) Notch2 integrates signaling by the transcription factors RBP-J and CREB1 to promote T cell cytotoxicity. Nat Immunol 9:1140–1147 18724371 10.1038/ni.1649

[CR36] Martin MD, Badovinac VP (2018) Defining memory CD8 T cell. Front Immunol 9:2692 30515169 10.3389/fimmu.2018.02692 PMC6255921

[CR37] Masopust D, Vezys V, Marzo AL, Lefrancois L (2001) Preferential localization of effector memory cells in nonlymphoid tissue. Science 291:2413–2417 11264538 10.1126/science.1058867

[CR38] Milner JJ, Nguyen H, Omilusik K, Reina-Campos M, Tsai M, Toma C, Delpoux A, Boland BS, Hedrick SM, Chang JT, Goldrath AW (2020) Delineation of a molecularly distinct terminally differentiated memory CD8 T cell population. Proc Natl Acad Sci USA 117:25667–25678 32978300 10.1073/pnas.2008571117 PMC7568335

[CR39] Moran AE, Holzapfel KL, Xing Y, Cunningham NR, Maltzman JS, Punt J, Hogquist KA (2011) T cell receptor signal strength in Treg and iNKT cell development demonstrated by a novel fluorescent reporter mouse. J Exp Med 208:1279–1289 21606508 10.1084/jem.20110308 PMC3173240

[CR78] Moran-Crusio K, Reavie L, Shih A, Abdel-Wahab O, Ndiaye-Lobry D, Lobry C, Figueroa ME, Vasanthakumar A, Patel J, Zhao X et al. (2011) Tet2 loss leads to increased hematopoietic stem cell self-renewal and myeloid transformation. Cancer Cell 20:11–24 10.1016/j.ccr.2011.06.001 PMC3194039 21723200

[CR40] Moskowitz DM, Zhang DW, Hu B, Le Saux S, Yanes RE, Ye Z, Buenrostro JD, Weyand CM, Greenleaf WJ, Goronzy JJ (2017) Epigenomics of human CD8 T cell differentiation and aging. Sci Immunol 2:eaag0192 28439570 10.1126/sciimmunol.aag0192 PMC5399889

[CR41] Oyake T, Itoh K, Motohashi H, Hayashi N, Hoshino H, Nishizawa M, Yamamoto M, Igarashi K (1996) Bach proteins belong to a novel family of BTB-basic leucine zipper transcription factors that interact with MafK and regulate transcription through the NF-E2 site. Mol Cell Biol 16:6083–6095 8887638 10.1128/mcb.16.11.6083 PMC231611

[CR42] Pais Ferreira D, Silva JG, Wyss T, Fuertes Marraco SA, Scarpellino L, Charmoy M, Maas R, Siddiqui I, Tang L, Joyce JA et al (2020) Central memory CD8(+) T cells derive from stem-like Tcf7(hi) effector cells in the absence of cytotoxic differentiation. Immunity 53:985–1000.e1011 33128876 10.1016/j.immuni.2020.09.005

[CR43] Palacios EH, Weiss A (2004) Function of the Src-family kinases, Lck and Fyn, in T-cell development and activation. Oncogene 23:7990–8000 15489916 10.1038/sj.onc.1208074

[CR44] Palmer DB (2013) The effect of age on thymic function. Front Immunol 4:316 24109481 10.3389/fimmu.2013.00316 PMC3791471

[CR45] Pipkin ME, Sacks JA, Cruz-Guilloty F, Lichtenheld MG, Bevan MJ, Rao A (2010) Interleukin-2 and inflammation induce distinct transcriptional programs that promote the differentiation of effector cytolytic T cells. Immunity 32:79–90 20096607 10.1016/j.immuni.2009.11.012 PMC2906224

[CR46] Pircher H, Moskophidis D, Rohrer U, Burki K, Hengartner H, Zinkernagel RM (1990) Viral escape by selection of cytotoxic T cell-resistant virus variants in vivo. Nature 346:629–633 1696684 10.1038/346629a0

[CR47] Renkema KR, Huggins MA, Borges da Silva H, Knutson TP, Henzler CM, Hamilton SE (2020) KLRG1(+) memory CD8 T cells combine properties of short-lived effectors and long-lived memory. J Immunol 205:1059–1069 32611727 10.4049/jimmunol.1901512 PMC7415731

[CR48] Richer MJ, Lang ML, Butler NS (2016) T cell fates zipped up: how the Bach2 basic leucine zipper transcriptional repressor directs T cell differentiation and function. J Immunol 197:1009–1015 27496973 10.4049/jimmunol.1600847 PMC4978142

[CR49] Rodriguez RM, Suarez-Alvarez B, Mosen-Ansorena D, Garcia-Peydro M, Fuentes P, Garcia-Leon MJ, Gonzalez-Lahera A, Macias-Camara N, Toribio ML, Aransay AM, Lopez-Larrea C (2015) Regulation of the transcriptional program by DNA methylation during human alphabeta T-cell development. Nucleic Acids Res 43:760–774 25539926 10.1093/nar/gku1340 PMC4333391

[CR50] Roychoudhuri R, Clever D, Li P, Wakabayashi Y, Quinn KM, Klebanoff CA, Ji Y, Sukumar M, Eil RL, Yu Z et al (2016) BACH2 regulates CD8(+) T cell differentiation by controlling access of AP-1 factors to enhancers. Nat Immunol 17:851–860 27158840 10.1038/ni.3441 PMC4918801

[CR51] Sallusto F, Lanzavecchia A, Araki K, Ahmed R (2010) From vaccines to memory and back. Immunity 33:451–463 21029957 10.1016/j.immuni.2010.10.008 PMC3760154

[CR52] Samelson LE (2002) Signal transduction mediated by the T cell antigen receptor: the role of adapter proteins. Annu Rev Immunol 20:371–394 11861607 10.1146/annurev.immunol.20.092601.111357

[CR53] Scharer CD, Barwick BG, Youngblood BA, Ahmed R, Boss JM (2013) Global DNA methylation remodeling accompanies CD8 T cell effector function. J Immunol 191:3419–3429 23956425 10.4049/jimmunol.1301395 PMC3800465

[CR54] Schauder DM, Shen J, Chen Y, Kasmani MY, Kudek MR, Burns R, Cui W (2021) E2A-regulated epigenetic landscape promotes memory CD8 T cell differentiation. Proc Natl Acad Sci USA 118:e2013452118 33859041 10.1073/pnas.2013452118 PMC8072256

[CR55] Sellars M, Huh JR, Day K, Issuree PD, Galan C, Gobeil S, Absher D, Green MR, Littman DR (2015) Regulation of DNA methylation dictates Cd4 expression during the development of helper and cytotoxic T cell lineages. Nat Immunol 16:746–754 26030024 10.1038/ni.3198 PMC4474743

[CR79] Shen L, Inoue A, He J, Liu Y, Lu F, Zhang Y (2014) Tet3 and DNA replication mediate demethylation of both the maternal and paternal genomes in mouse zygotes. Cell Stem Cell 15:459–471 10.1016/j.stem.2014.09.002 PMC4201500 25280220

[CR56] Smith-Garvin JE, Burns JC, Gohil M, Zou T, Kim JS, Maltzman JS, Wherry EJ, Koretzky GA, Jordan MS (2010) T-cell receptor signals direct the composition and function of the memory CD8+ T-cell pool. Blood 116:5548–5559 20847203 10.1182/blood-2010-06-292748 PMC3031403

[CR57] Solouki S, Huang W, Elmore J, Limper C, Huang F, August A (2020) TCR signal strength and antigen affinity regulate CD8(+) memory T cells. J Immunol 205:1217–1227 32759295 10.4049/jimmunol.1901167 PMC8104072

[CR58] Soto-Heredero G, Gomez de Las Heras MM, Escrig-Larena JI, Mittelbrunn M (2023) Extremely differentiated T cell subsets contribute to tissue deterioration during aging. Annu Rev Immunol 41:181–205 37126417 10.1146/annurev-immunol-101721-064501

[CR59] Teghanemt A, Misel-Wuchter K, Heath J, Thurman A, Pulipati P, Dixit G, Geesala R, Meyerholz DK, Maretzky T, Pezzulo A, Issuree PD (2023) DNA demethylation fine-tunes IL-2 production during thymic regulatory T cell differentiation. EMBO Rep. 24:e55543 36880575 10.15252/embr.202255543 PMC10157375

[CR60] Toumi R, Yuzefpolskiy Y, Vegaraju A, Xiao H, Smith KA, Sarkar S, Kalia V (2022) Autocrine and paracrine IL-2 signals collaborate to regulate distinct phases of CD8 T cell memory. Cell Rep. 39:110632 35417685 10.1016/j.celrep.2022.110632 PMC12948409

[CR61] Tsagaratou A, Aijo T, Lio CW, Yue X, Huang Y, Jacobsen SE, Lahdesmaki H, Rao A (2014) Dissecting the dynamic changes of 5-hydroxymethylcytosine in T-cell development and differentiation. Proc Natl Acad Sci USA 111:E3306–3315 25071199 10.1073/pnas.1412327111 PMC4136618

[CR62] Tsagaratou A, Gonzalez-Avalos E, Rautio S, Scott-Browne JP, Togher S, Pastor WA, Rothenberg EV, Chavez L, Lahdesmaki H, Rao A (2017) TET proteins regulate the lineage specification and TCR-mediated expansion of iNKT cells. Nat Immunol 18:45–53 27869820 10.1038/ni.3630 PMC5376256

[CR63] Turner R, Tjian R (1989) Leucine repeats and an adjacent DNA binding domain mediate the formation of functional cFos-cJun heterodimers. Science 243:1689–1694 2494701 10.1126/science.2494701

[CR64] Vaisvila R, Ponnaluri VKC, Sun Z, Langhorst BW, Saleh L, Guan S, Dai N, Campbell MA, Sexton BS, Marks K et al (2021) Enzymatic methyl sequencing detects DNA methylation at single-base resolution from picograms of DNA. Genome Res 31:1280–1289 34140313 10.1101/gr.266551.120 PMC8256858

[CR65] van de Wall S, Badovinac VP, Harty JT (2021) Influenza-specific lung-resident memory CD8(+) T cells. Cold Spring Harb Perspect Biol 13:a037978 33288540 10.1101/cshperspect.a037978 PMC7849341

[CR66] Vierbuchen T, Ling E, Cowley CJ, Couch CH, Wang X, Harmin DA, Roberts CWM, Greenberg ME (2017) AP-1 transcription factors and the BAF complex mediate signal-dependent enhancer selection. Mol Cell 68:1067–1082.e1012 29272704 10.1016/j.molcel.2017.11.026 PMC5744881

[CR67] Walters RD, Drullinger LF, Kugel JF, Goodrich JA (2013) NFATc2 recruits cJun homodimers to an NFAT site to synergistically activate interleukin-2 transcription. Mol Immunol 56:48–56 23665382 10.1016/j.molimm.2013.03.022 PMC3686915

[CR68] Wang D, Diao H, Getzler AJ, Rogal W, Frederick MA, Milner J, Yu B, Crotty S, Goldrath AW, Pipkin ME (2018) The transcription factor Runx3 establishes chromatin accessibility of cis-regulatory landscapes that drive memory cytotoxic T lymphocyte formation. Immunity 48:659–674.e656 29669249 10.1016/j.immuni.2018.03.028 PMC6750808

[CR69] Wherry EJ, Teichgraber V, Becker TC, Masopust D, Kaech SM, Antia R, von Andrian UH, Ahmed R (2003) Lineage relationship and protective immunity of memory CD8 T cell subsets. Nat Immunol 4:225–234 12563257 10.1038/ni889

[CR70] Wu X, Zhang Y (2017) TET-mediated active DNA demethylation: mechanism, function and beyond. Nat Rev Genet 18:517–534 28555658 10.1038/nrg.2017.33

[CR71] Xie Z, Bailey A, Kuleshov MV, Clarke DJB, Evangelista JE, Jenkins SL, Lachmann A, Wojciechowicz ML, Kropiwnicki E, Jagodnik KM et al (2021) Gene set knowledge discovery with Enrichr. Curr Protoc 1:e90 33780170 10.1002/cpz1.90 PMC8152575

[CR72] Xue S, Liu C, Sun X, Li W, Zhang C, Zhou X, Lu Y, Xiao J, Li C, Xu X et al (2016) TET3 Inhibits Type I IFN Production Independent of DNA Demethylation. Cell Rep. 16:1096–1105 27425624 10.1016/j.celrep.2016.06.068

[CR73] Youngblood B, Hale JS, Kissick HT, Ahn E, Xu X, Wieland A, Araki K, West EE, Ghoneim HE, Fan Y et al (2017) Effector CD8 T cells dedifferentiate into long-lived memory cells. Nature 552:404–409 29236683 10.1038/nature25144 PMC5965677

[CR74] Yukawa M, Jagannathan S, Vallabh S, Kartashov AV, Chen X, Weirauch MT, Barski A (2020) AP-1 activity induced by co-stimulation is required for chromatin opening during T cell activation. J Exp Med 217:e20182009 31653690 10.1084/jem.20182009 PMC7037242

[CR75] Zhang X, Su J, Jeong M, Ko M, Huang Y, Park HJ, Guzman A, Lei Y, Huang YH, Rao A et al (2016) DNMT3A and TET2 compete and cooperate to repress lineage-specific transcription factors in hematopoietic stem cells. Nat Genet 48:1014–1023 27428748 10.1038/ng.3610 PMC4957136

[CR76] Zhou X, Yu S, Zhao DM, Harty JT, Badovinac VP, Xue HH (2010) Differentiation and persistence of memory CD8(+) T cells depend on T cell factor 1. Immunity 33:229–240 20727791 10.1016/j.immuni.2010.08.002 PMC2928475

[CR77] Zikherman J, Parameswaran R, Weiss A (2012) Endogenous antigen tunes the responsiveness of naive B cells but not T cells. Nature 489:160–164 22902503 10.1038/nature11311 PMC3438375

