# Supplementary material for: Developmental epigenetic programming by Tet1/3 determines peripheral CD8 T cell fate
Source: EMBO Rep. 2025 Apr 2;26(10):2494–518. doi: 10.1038/s44319-025-00439-z (PMC12116802; doi:10.1038/s44319-025-00439-z)
Supplement: Supplementary file 13 — Expanded View Figures [file 44319_2025_439_MOESM13_ESM.pdf]

Expanded View Figures

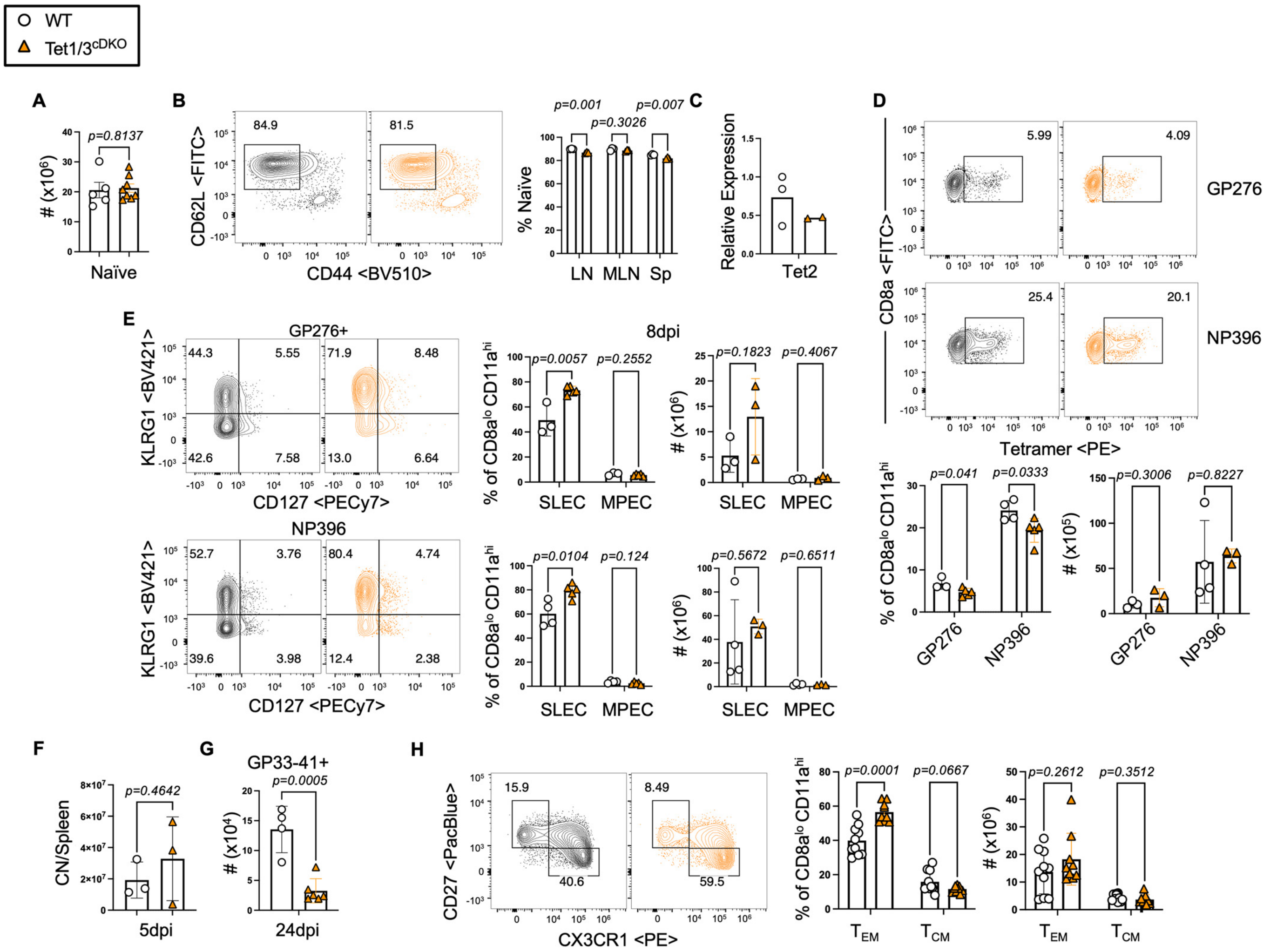

Figure EV1. Tet1 and Tet3 deficiency results in increased SLECs and effector memory T-cell differentiation during acute LCMV infection.

(**A**) Absolute numbers of mature CD8 + T cells from control Tet1/3fl/fl and Rorc(t)CreTgTet1/3fl/fl mice. (WT = 5, Tet1/3^{cDKO} = 8 biological replicates). Data are mean ± SEM, unpaired *t* test. (**B**) Representative FACS plots of CD62L and CD44 expression on CD8 T cells and frequency of naive CD8 T cells from indicated secondary lymphoid organs of control P14 Tet1/3fl/fl and P14-Rorc(t)CreTgTet1/3fl/fl mice, pregated on LiveTCRb+CD8a+ cells. (WT = 3, Tet1/3^{cDKO} = 3 biological replicates). Data are mean ± SEM, multiple unpaired *t* tests. (**C**) Relative expression of Tet2 normalized to HPRT in naive Tet1/3fl/fl and Rorc(t)CreTgTet1/3fl/fl CD8 T cells. (WT = 3, Tet1/3^{cDKO} = 2 biological replicates). (**D**) Representative FACS plots and quantification of frequency and number of GP276- and NP396-tetramer+ CD8 T cells. (WT = 3-4, Tet1/3^{cDKO} = 5 biological replicates) Data are mean ± SEM, multiple unpaired *t* tests. (**E**) Representative FACS plots and quantification of frequency and number of SLEC and MPECs among GP276-tetramer+ and NP396-tetramer+ CD8 cells. (WT = 3-4, Tet1/3^{cDKO} = 5 biological replicates) Data are mean ± SEM, multiple unpaired *t* tests. (**F**) RT-qPCR assessment of LCMV copy number (CN) per spleen 5dpi. (WT = 3, Tet1/3^{cDKO} = 3 biological replicates) Data are mean ± SEM, unpaired *t* test. (**G**) Absolute number of GP33-tetramer+ CD8 T cells 24dpi. (WT = 4, Tet1/3^{cDKO} = 6 biological replicates). Data are mean ± SEM, unpaired *t* test. (**H**) Representative FACS and quantification of frequency and number of TEM/TCM 8dpi. (WT = 10, Tet1/3^{cDKO} = 9 biological replicates). Data are mean ± SEM, multiple unpaired *t* tests. Data information: *P* values are indicated in the figures and $P < 0.05$ was considered significant; paired male or female mice were used and no gender biases associated with genotypes were observed. (**A**, **B**, **H**) Experiments were replicated at least three times. Source data are available online for this figure.

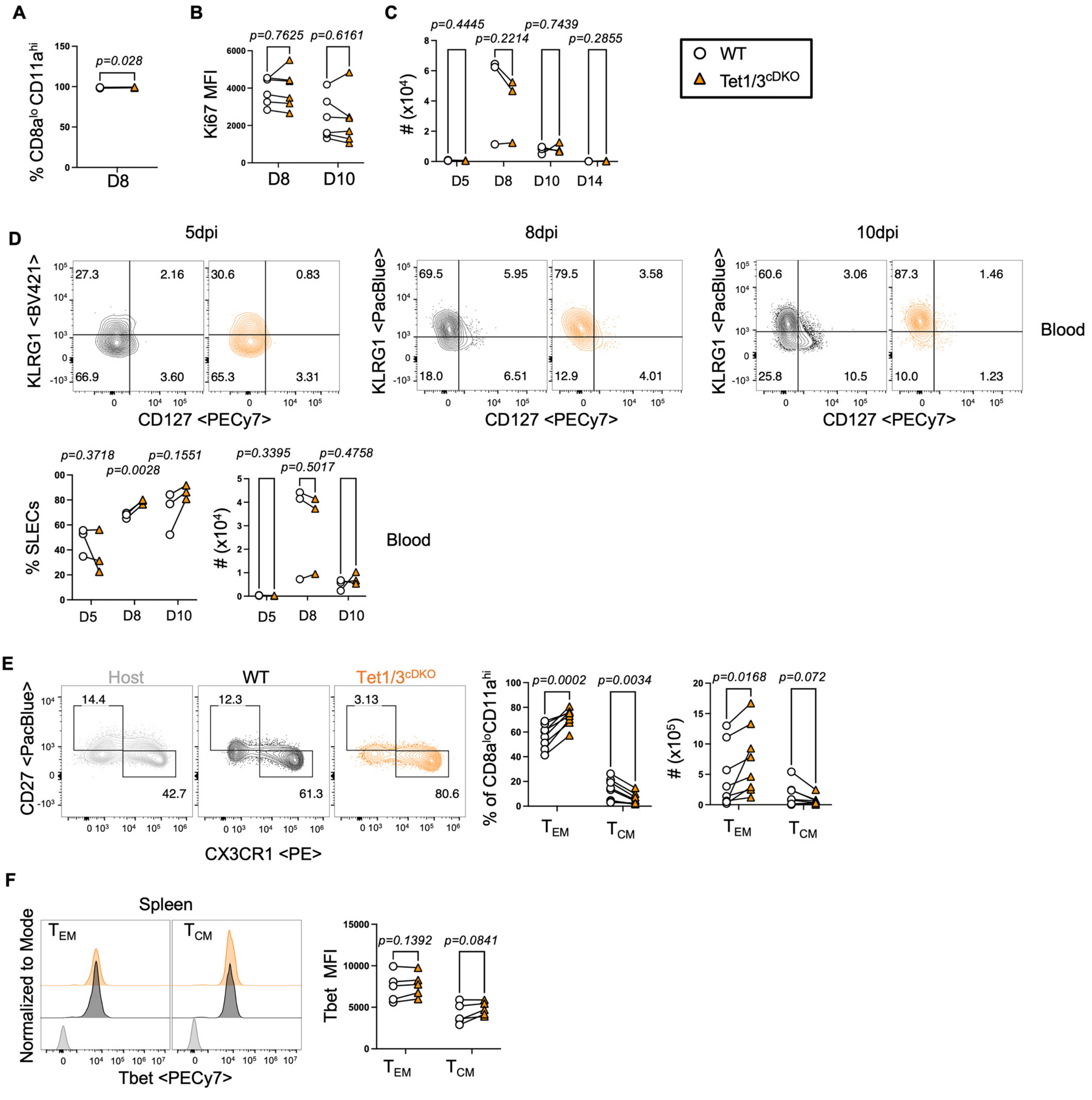

Figure EV2. Tet1/3 restrain the differentiation of SLECs and effector memory T cells in a cell-intrinsic manner.

(**A**) Percent of CD8a^{lo}CD11a^{hi} cells among transferred P14 CD8 T cells 8 dpi. (WT = 5, Tet1/3^{cDKO} = 5 biological replicates) Significance was determined by paired *t* test. (**B**) Ki67 MFI among transferred P14 CD8 T cells in the blood at 8 and 10 dpi. (WT = 5, Tet1/3^{cDKO} = 5 biological replicates) Significance was determined by multiple paired t tests. (**C**) Absolute numbers of transferred P14 CD8 T cells in the blood at indicated time points. (WT = 3, Tet1/3^{cDKO} = 3 biological replicates) Significance was determined by multiple paired *t* tests. (**D**) Representative FACS plots and quantification of frequency and number of SLECs recovered at indicated time points in the blood. (WT = 3, Tet1/3^{cDKO} = 3 biological replicates) Significance was determined by multiple paired *t* tests. (**E**) Representative FACS plots and quantification of frequency and number of TEM/TCM cells recovered 8 dpi. (WT = 8, Tet1/3^{cDKO} = 8 biological replicates) Significance was determined by multiple paired *t* tests. (**F**) Representative histograms and quantification of Tbet expression in TEM/TCM populations. (WT = 5, Tet1/3^{cDKO} = 5 biological replicates) Significance was determined by multiple paired *t* tests. Data information: *P* values are indicated in the figures and $P < 0.05$ was considered significant; paired male or female mice were used and no gender biases associated with genotypes were observed. (**A–D**) Experiments were replicated at least three times. Source data are available online for this figure.

 © The Author(s)

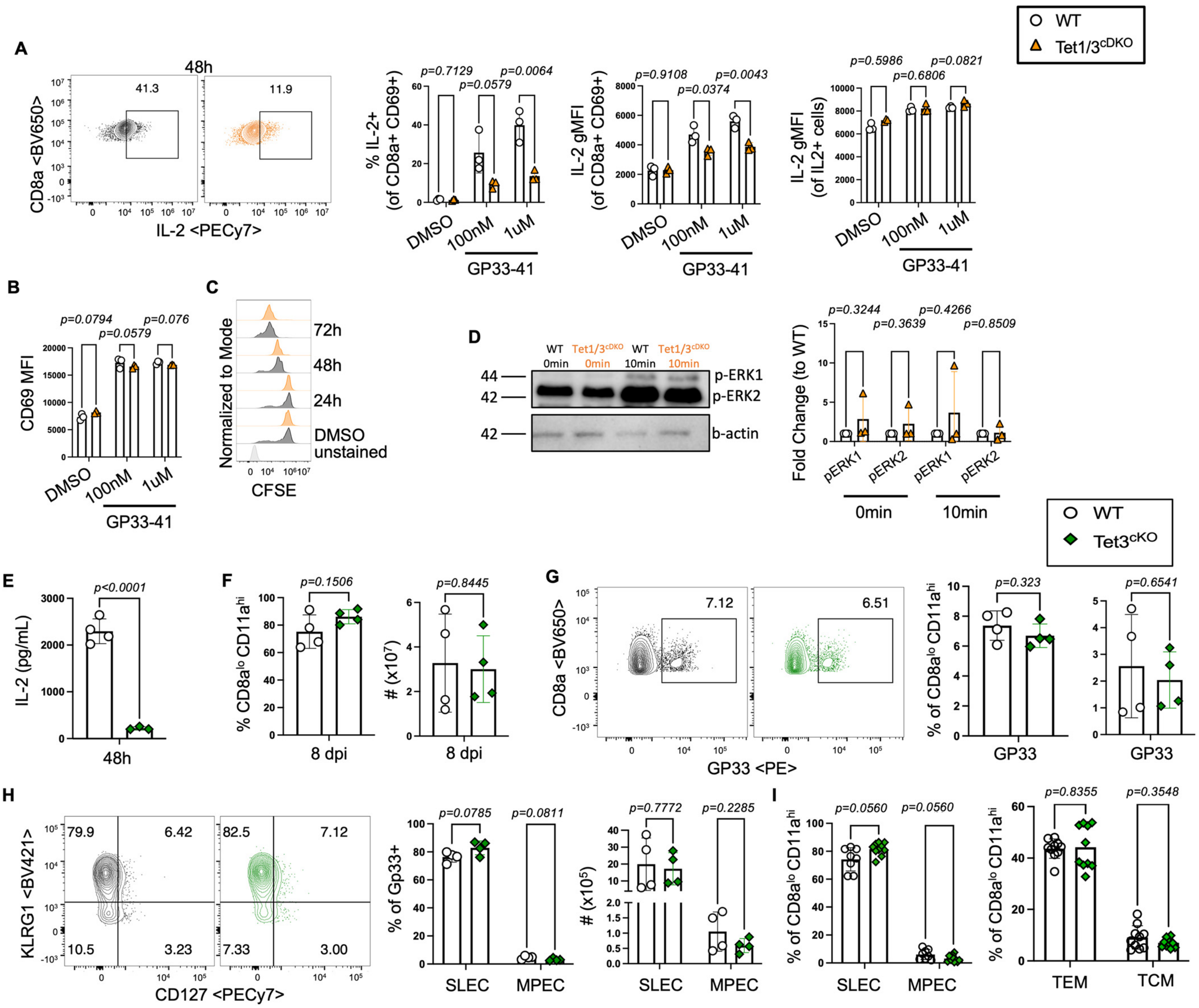

Figure EV3. Tet1/3 restrict SLEC/T_{EM} CD8 T-cell differentiation by controlling TCR-dependent epigenetic circuits.

(**A–C**) Naive P14 WT and Tet1/3^{cDKO} splenocytes were stimulated in vitro with gp33-41 peptide and anti-CD28 for indicated time points. (**D**) FACS-Sorted naive WT and Tet1/3^{cDKO} T cells were stimulated in vitro via anti-CD3 and anti-CD28 for indicated time points. (**A**) Representative FACS plots and quantification of frequency of IL-2+ cells among CD69+ cells, gMFI of IL-2 among CD69+ cells, gMFI of IL-2 among IL-2^{+} cells, 48-h post activation. Cells were treated with Brefeldin A and Monensin for the last 5 h of stimulation (WT = 3, Tet1/3^{cDKO} = 3 biological replicates). Data are mean ± SEM, multiple unpaired *t* tests. (**B**) CD69 MFI on CD8 T cells 48-h post activation (WT = 3, Tet1/3^{cDKO} = 3 biological replicates). Data are mean ± SEM, multiple unpaired *t* test. (**C**) Representative histogram showing CD8 T-cell proliferation by CFSE dilution. (**D**) Representative immunoblot and quantification of phospho-ERK1/2. Blots were quantified using ImageJ and normalized to show quantification of three independent experiments. Data are mean ± SEM, multiple unpaired *t* tests. (**E**) Quantification of IL-2 levels in the supernatants of activated CD8 T cells by ELISA. (WT = 4, Tet3^{cKO} = 4 biological replicates). Data are mean ± SEM, multiple unpaired *t* tests. (**F**) Percentage and number of antigen-experienced CD8 T cells 8dpi. (WT = 4, Tet3^{cKO} = 4 biological replicates) Data are mean ± SEM, unpaired *t* test. (**G**) Representative FACS plots and quantification of frequency and number of GP33-tetramer+ CD8 T cells. Cells were pregated on LiveTCRβ^{+}CD8a^{lo} CD11a^{hi} cells. (WT = 4, Tet3^{cKO} = 4 biological replicates) Data are mean ± SEM, unpaired *t* test. (**H**) Representative FACS plots and quantification of frequency and number of SLECs/MPECs among GP33-tetramer+ cells 8 dpi. (WT = 4, Tet3^{cKO} = 4 biological replicates) Data are mean ± SEM, multiple unpaired *t* tests. (**I**) Quantification of frequency of SLECs/MPECs among activated CD8 T cells 8 dpi. Data is a summary of 2 independent experiments (WT = 8, Tet3^{cKO} = 10 biological replicates) Data are mean ± SEM, multiple unpaired *t* tests. Data information: *P* values are indicated in the figures and $P < 0.05$ was considered significant; paired male or female mice were used and no gender biases associated with genotypes were observed. (**A**) Experiments were replicated three times. (**B–D**) Experiments were replicated twice. Source data are available online for this figure.

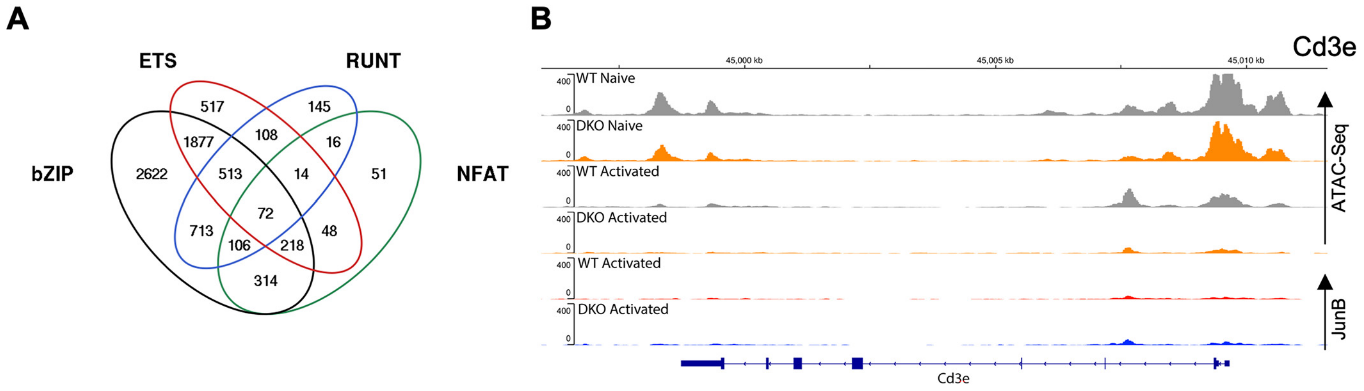

Figure EV4. Loss of Tet1 and Tet3 impairs chromatin accessibility at regulatory regions in TCR-response genes.

(**A**) Overlap of differentially accessible peaks containing bZIP, ETS, Runt or NFAT in P14 Tet1/3^{cDKO} and WT CD8 T cells isolated 5dpi with LCMV-Armstrong (**B**) Integrated genome browser view (IGV) shots of the Cd3e gene. Tracks show the reference gene, ATAC-Seq peaks (WT in gray, Tet1/3^{cDKO} in orange) and JunB Cut and Run peaks (WT in red and Tet1/3^{cDKO} in blue) in naive or activated cells.

 © The Author(s)

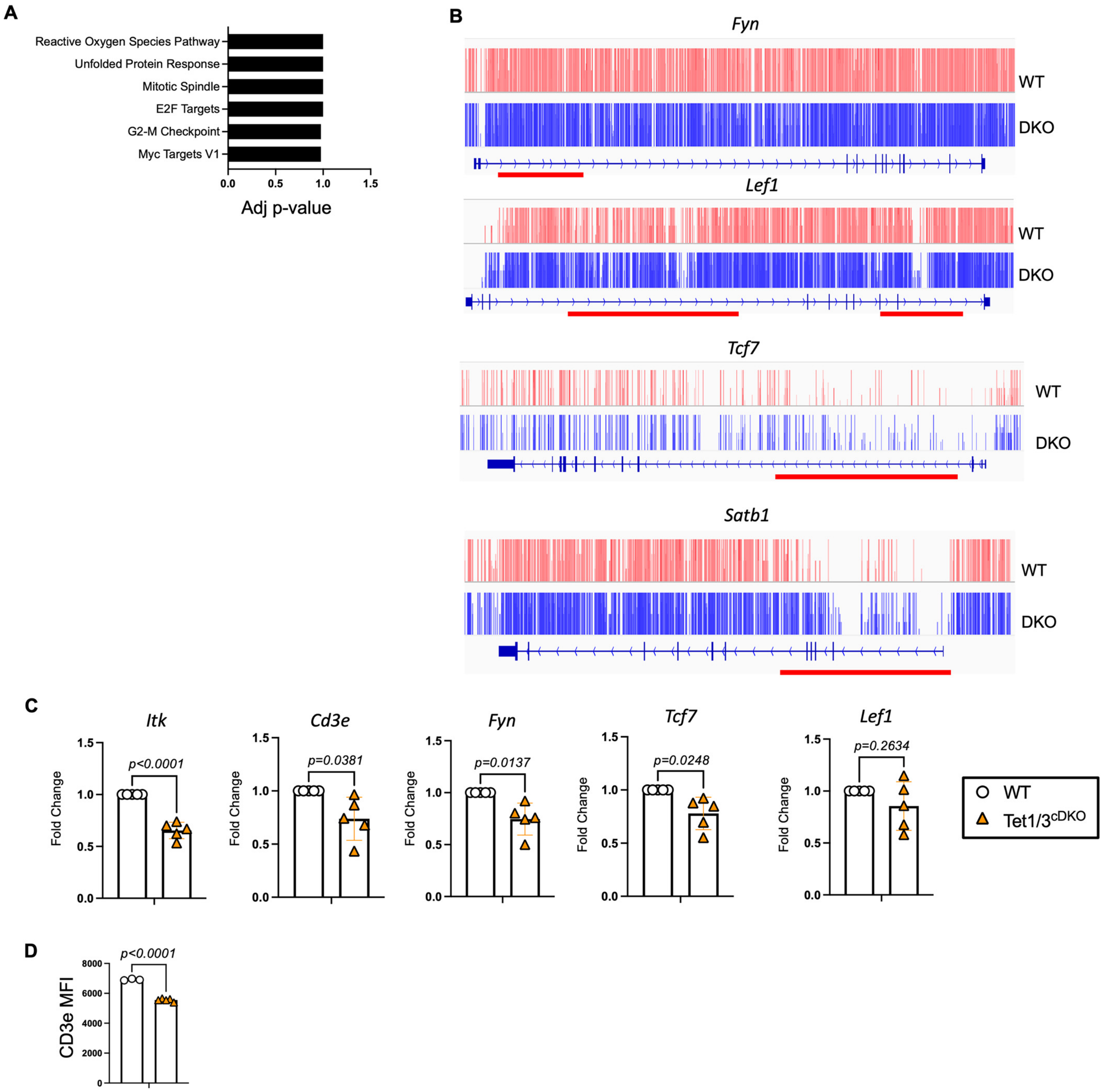

Figure EV5. CD8 T cells undergo Tet/3-dependent DNA demethylation during thymic development.

(**A**) Gene enrichment analysis of differentially methylated gene loci between WT activated and naive CD8 T cells. Adjusted *P* value was calculated using the Benjamini–Hochberg method. (**B**) Integrated genome browser view (IGV) shots of the Fyn, Lef1, Tcf7 and Satb1 gene loci. Tracks show the reference gene, and EM-Seq methylation peaks (WT in red and Tet1/3^{cDKO} in blue) in activated CD8 T cells. Red bar indicates regions with differentially methylated CpGs. (**C**) Fold change of Itk, Cd3e, Fyn, Tcf7 and Lef1 expression in activated CD8 T cells by RT-qPCR. Data is normalized to HPRT. (WT = 5, Tet1/3^{cDKO} = 5 biological replicates). Cells were activated in vitro for 48 h with anti-CD3/CD28. Data are mean ± SEM, unpaired *t* test. (**D**) CD3e MFI 48 h post activation with anti-CD3/CD28. (WT = 3, Tet1/3^{cDKO} = 5 biological replicates). Data are mean ± SEM, unpaired t test. Data information: *P* values are indicated in the figures and $P < 0.05$ was considered significant; paired male or female mice were used and no gender biases associated with genotypes were observed. (**A**) Experiments were replicated three times. (**C**, **D**) Experiments were replicated at least three times. Source data are available online for this figure.

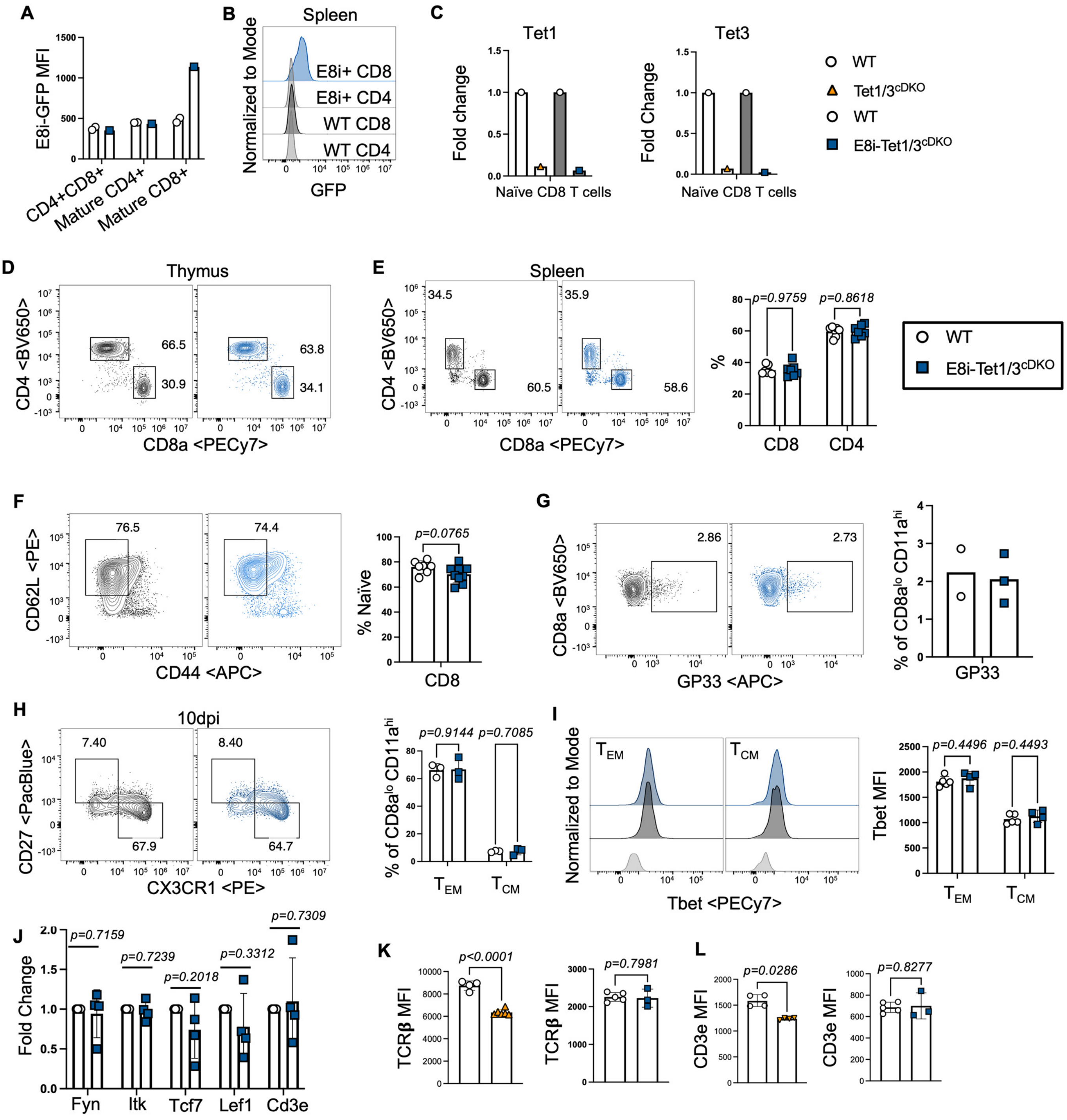
A
E8i-GFP MFI
CD4+CD8+
Mature CD4+
Mature CD8+
B
Spleen
Normalized to Mode
E8i+ CD8
E8i+ CD4
WT CD8
WT CD4
GFP
C
Tet1
Tet3
Fold change
Fold Change
Naïve CD8 T cells
WT
Tet1/3cDKO
WT
E8i-Tet1/3cDKO
D
Thymus
CD4 <BV650>
CD8a <PECy7>
66.5
30.9
63.8
34.1
E
Spleen
34.5
60.5
35.9
58.6
p=0.9759
p=0.8618
CD8
CD4
WT
E8i-Tet1/3cDKO
F
CD62L <PE>
CD44 <APC>
76.5
74.4
p=0.0765
% Naïve
G
CD8a <BV650>
GP33 <APC>
2.86
2.73
% of CD8alo CD11ahi
GP33
H
10dpi
CD27 <PacBlue>
CX3CR1 <PE>
7.40
67.9
8.40
64.7
p=0.9144
p=0.7085
TEM
TCM
I
Normalized to Mode
Tbet <PECy7>
p=0.4496
p=0.4493
Tbet MFI
J
Fold Change
p=0.7159
p=0.7239
p=0.2018
p=0.3312
p=0.7309
Fyn
Itk
Tcf7
Lef1
Cd3e
K
p<0.0001
p=0.7981
TCRβ MFI
L
p=0.0286
p=0.8277
CD3e MFI

© The Author(s)

Figure EV6. Tet1/3 are dispensable in peripheral CD8 T cells for effector and memory differentiation.

(**A**) GFP MFI expressed in control Tet1/3fl/fl and E8iCreTgTet1/3fl/fl thymic populations. (WT = 2, Tet1/3^{cDKO} = 2 biological replicates). (**B**) Representative histograms showing GFP expression in control Tet1/3fl/fl- and E8iCreTgTet1/3fl/fl lymphocytes from the spleen (**C**) Fold change of Tet1 and Tet3 genomic copies in naive CD8 T cells from control Tet1/3fl/fl, Rorc(t)CreTgTet1/3fl/fl and E8iCreTgTet1/3fl/fl mice. (**D**) Representative FACS plots of CD4 and CD8 thymic T-cell populations from control Tet1/3fl/fl and E8iCreTgTet1/3fl/fl mice. Cells were pregated on LiveCD24-TCRb+CD69- cells. (**E**) Representative FACS plots and frequency of peripheral CD4 and CD8 splenic T-cell populations from control Tet1/3fl/fl and E8iCreTgTet1/3fl/fl mice. Cells were pregated on LiveTCRb+ cells. (WT = 7, Tet1/3^{cDKO} = 7 biological replicates). Data are mean ± SEM, multiple unpaired *t* tests. (**F**) Representative FACS plots and frequency of naive CD8 T cells from the lymph nodes and spleens of control Tet1/3fl/fl and E8iCreTgTet1/3fl/fl mice. Cells were pregated on LiveTCRb+CD8a+ cells. (WT = 7, Tet1/3^{cDKO} = 9 biological replicates). Data are mean ± SEM, unpaired t test. (**G**) Representative histograms and quantification of GP33-tetramer+ CD8 T cells 8 dpi. (WT = 2, Tet1/3^{cDKO} = 3 biological replicates). (**H**) Representation flow plots and quantification of TEM and TCM populations in the spleen 10 dpi. (WT = 3, Tet1/3^{cDKO} = 3 biological replicates) Data are mean ± SEM, multiple unpaired *t* tests. (**I**) Representative histograms and quantification of Tbet expression in TEM/TCM populations 30 dpi. (WT = 5, Tet1/3^{cDKO} = 4 biological replicates) Data are mean ± SEM, multiple unpaired tests. (**J**) Fold change of Fyn, Itk, Tcf, Lef1, Cd3e mRNA expression in activated CD8 T cells from control and E8iCreTgTet1/3fl/fl mice. Data is a summary of 2 independent experiments (WT = 4, Tet1/3cDKO = 4 biological replicates) Data are mean ± SEM, multiple unpaired *t* tests. (**K**) TCRb MFI on bulk CD8 + T cells from the spleen 8 dpi with LCMV-Armstrong. Data are mean ± SEM, unpaired *t* test. (WT = 4, RorcCreTet1/3^{cDKO} = 6 biological replicates; WT = 5, E8iCre-Tet1/3 ^{cDKO} = 3 biological replicates) Data are mean ± SEM, unpaired *t* test. (**L**) Cd3e MFI on bulk CD8 + T cells from the spleen 10 dpi with LCMV-Armstrong. Data are mean ± SEM, unpaired *t* test. (WT = 4, RorcCreTet1/3^{cDKO} = 4 biological replicates; WT = 5, E8iCre-Tet1/3 ^{cDKO} = 3 biological replicates) Data are mean ± SEM, unpaired *t* test. Data information: *P* values are indicated in the figures and $P < 0.05$ was considered significant; paired male or female mice were used and no gender biases associated with genotypes were observed. (**A**, **B**, **D**–**L**) Experiments were replicated at least two times. Source data are available online for this figure.
